# Supplementary material for: Blockage of autophagy causes severe skeletal muscle disruption in a mouse model for myofibrillar myopathy 6
Source: Nat Commun. 2026 Apr 11;17:3436. doi: 10.1038/s41467-026-71749-6 (PMC13076893; doi:10.1038/s41467-026-71749-6)

Supplemental material for

Blockage of autophagy causes severe skeletal muscle disruption in a mouse model for myofibrillar myopathy 6

Kerstin Filippi, Kathrin Graf-Riesen, Maithreyan Kuppusamy, Andreas Unger, Kenichi Kimura, Martin Matijass, Henrique Baeta, Magdalena Podlacha, Daniel Haertter, Alexei P. Kudin, Martin Wiemann, Grzegorz Węgrzyn, Cornelia Kornblum, Jens Reimann, Wolfgang A. Linke, Pitter F. Huesgen, Wolfram S. Kunz, Bernd K. Fleischmann, Michael Hesse

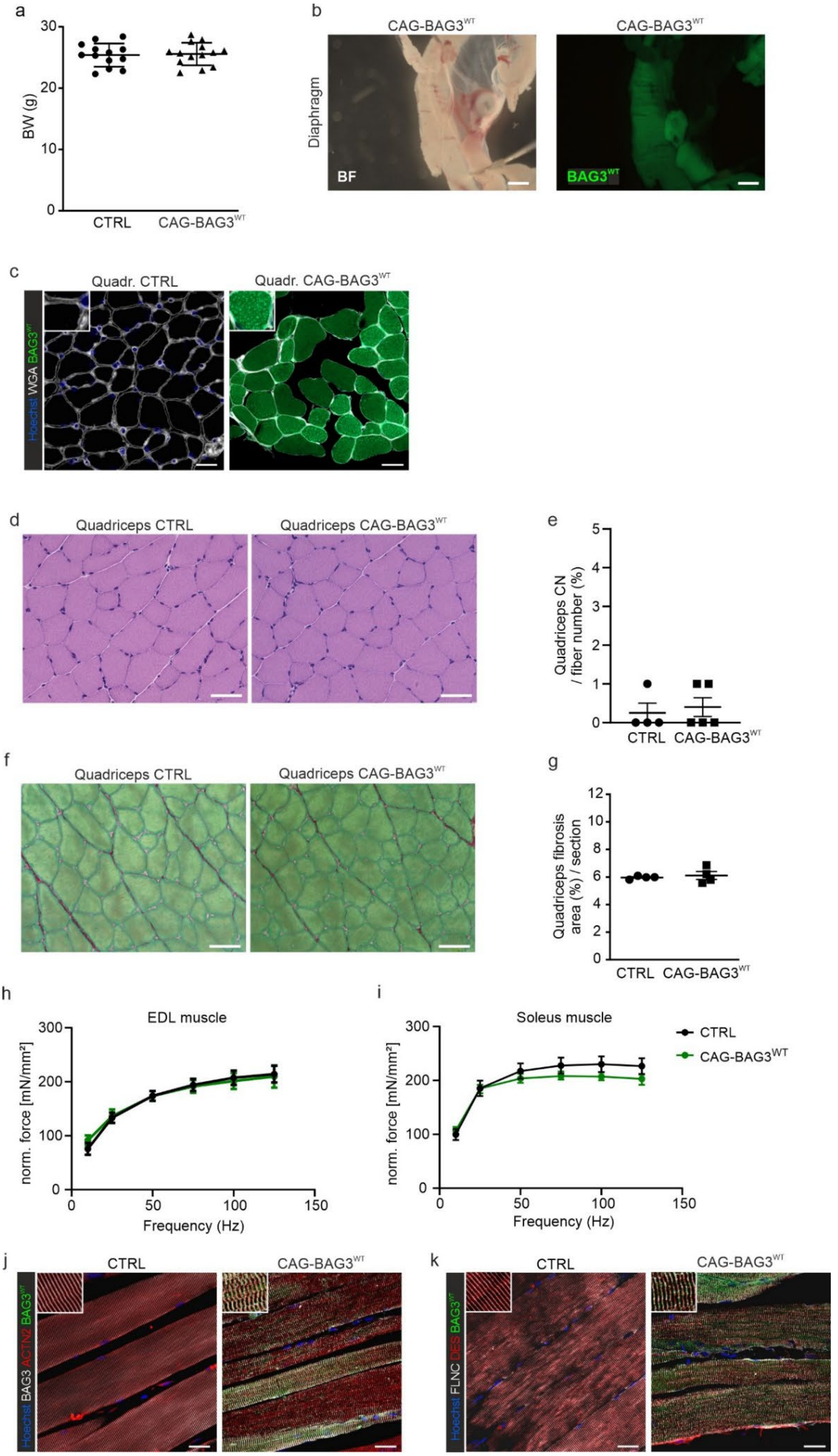

Supplementary Figure 1. CAG-BAG3^{WT} control mouse line displays no obvious phenotype

(a) Body weight of CTRL (n = 13) and CAG-BAG3^{WT}-mice (n = 14) at 5-weeks. Mean ± SEM. Statistical test = two-sided unpaired Student's t-test. (b) Homogenous BAG3^{P209L}-eGFP expression in diaphragm from CAG-BAG3^{WT}-mice. BF=bright field, Scale bar = 1000 µm; (c) Strong eGFP fluorescence in cross-sections from Quadriceps muscle from CAG-BAG3^{P209L}-mice. Scale bar = 50 µm. (d) Hematoxylin-eosin staining demonstrated no centralization of myocyte cell nuclei in quadriceps muscle from 4 weeks old CAG-BAG3^{WT}-mice. Scale bar = 50 µm. (e) Quantification revealed no difference of the number of centralized nuclei in Quadriceps muscle of CAG-BAG3^{WT}-mice (n = 5 biologically independent CAG-BAG3^{WT} mice; n = 4 biologically independent CTRL mice), shown is mean ± SEM, statistical test = two-sided unpaired Student's t-test. (f) Sirius Red-staining with Fast Green counterstain of Quadriceps muscle from 4 weeks old CAG-BAG3^{WT} and CTRL mice. Scale bars = 50 µm. (g) Quantification of fibrosis in Quadriceps muscle from (f) (n = 4 biologically independent mice per group); shown is mean ± SEM, statistical test = two-sided unpaired Student's t-test. (h,i) EDL and M. soleus of four-week-old CAG-BAG3^{WT} mice develop comparable normalized force as control mice during frequency-dependent force measurement (FFR). Sol.: n = 6 biologically independent mice per group; EDL: n = 7 biologically independent CAG-BAG3^{WT} mice; n = 11 biologically independent CTRL mice; shown is mean ± SEM, statistical test = two-way repeated-measures ANOVA, followed by Šídák's multiple-comparisons test. (j,k) Tissue of the Quadriceps muscle of controls (CTRL) and CAG-BAG3^{WT} mice showed distinct cross-striation (arrows in inserts) after staining for (j) BAG3, α-actinin, and (k) desmin and FLNC. Scale bars = 25 µm. (a-g,j,k) Experiments were repeated three times using biologically independent replicates, yielding similar results. Source data are provided as a Source Data file.

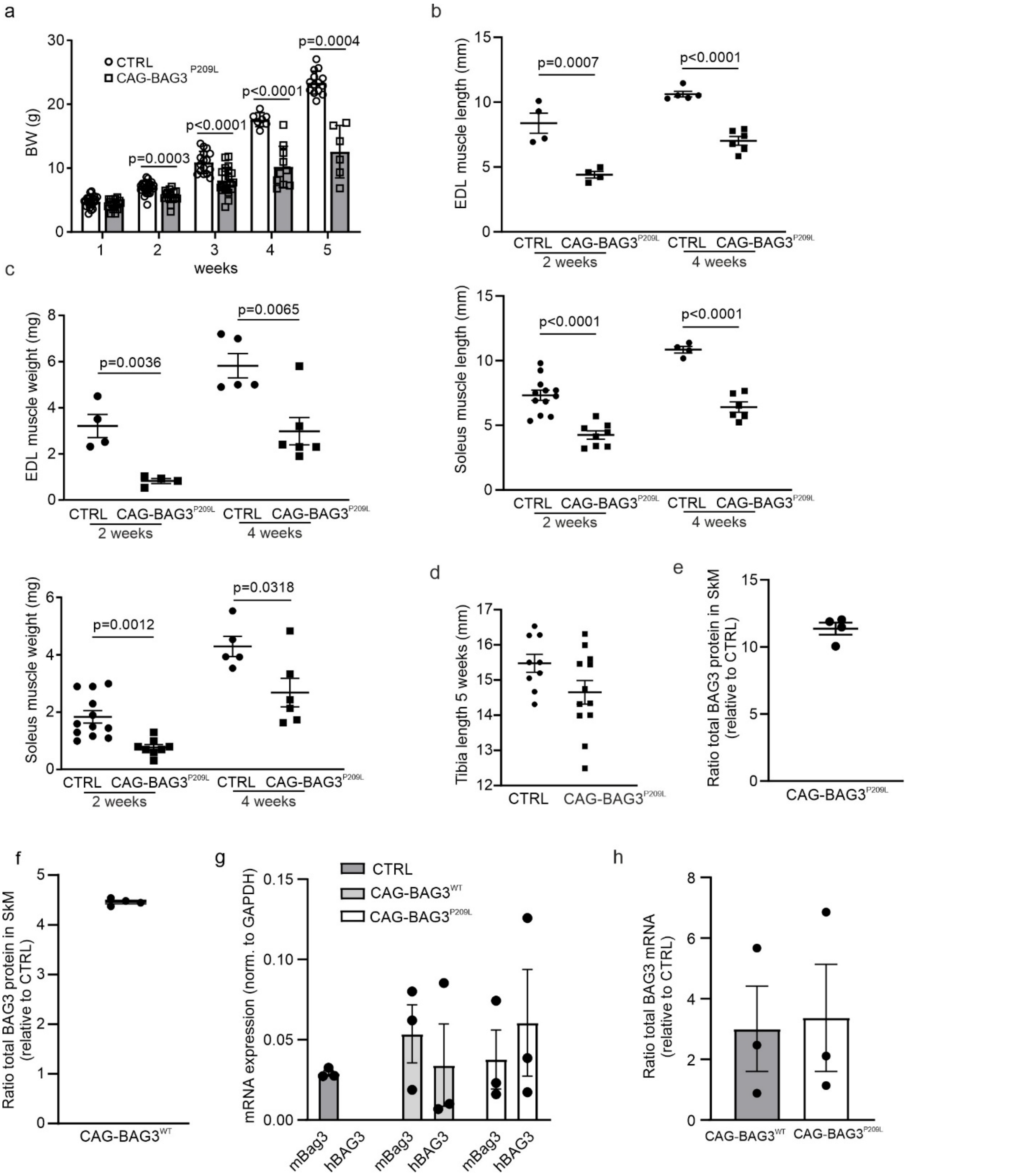

Supplementary Figure 2. Muscle weight, Satellite Cells, and centralized nuclei in CAG-BAG3^{P209L}-mice

(a) Growth curve of CTRL (W1: n = 23/ W2: n = 21/ W3: n = 16/ W4: n = 7/ W5: n = 14 CTRL-mice) and CAG-BAG3^{P209L}-mice (W1: n = 17/ W2: n = 15/ W3: n = 21/ W4: n = 10/ W5: n = 6 CAG-BAG3^{P209L}-mice) from 1 to 5-weeks. Mean ± SEM. Two-sided unpaired Student's t-test. Length (b) and weight (c) (W2 EDL: n = 4/ W4 EDL: n = 6/ W2 Sol.: n = 8/ W4 Sol.: n = 6 CAG-BAG3^{P209L}-mice; W2 EDL: n = 4/ W4 EDL: n = 5/ W2 Sol.: n = 12/ W4 Sol.: n = 5 CTRL-mice) of EDL and soleus muscles were significantly reduced in two and four weeks old CAG-BAG3^{P209L}-mice when compared with controls. Mean ± SEM. Two-sided unpaired Student's t-test. (d) Tibia length of 5 weeks old CAG-BAG3^{P209L} (n = 12 mice) and CTRL-mice (n = 9 mice). Mean ± SEM. Two-sided unpaired Student's t-test. (a-d) n denotes the number of biologically independent mice. (e-f) Ratio of total BAG3 protein

levels in skeletal muscle (SkM) of CAG-BAG3^{P209L}-mice (e) and CAG-BAG3^{WT}-mice (f) relative to CTRL (n = 4 biologically independent mice per group). Data are shown as mean ± SEM. (g) mRNA expression of mouse (mBag3) and human BAG3 (hBAG3) normalized to GAPDH in control (CTRL), CAG-BAG3^{WT}, and CAG-BAG3^{P209L} groups (n = 3 biologically independent mice per group). Mean ± SEM, statistical test = Two-way repeated-measures ANOVA with Tukey's multiple comparisons test. (h) Quantification of total BAG3 mRNA levels relative to CTRL shows no significant differences between CAG-BAG3^{WT} and CAG-BAG3^{P209L} (n = 3 biologically independent mice per group). Mean ± SEM, statistical test = two-sided unpaired Student’s t-test. Source data are provided as a Source Data file.

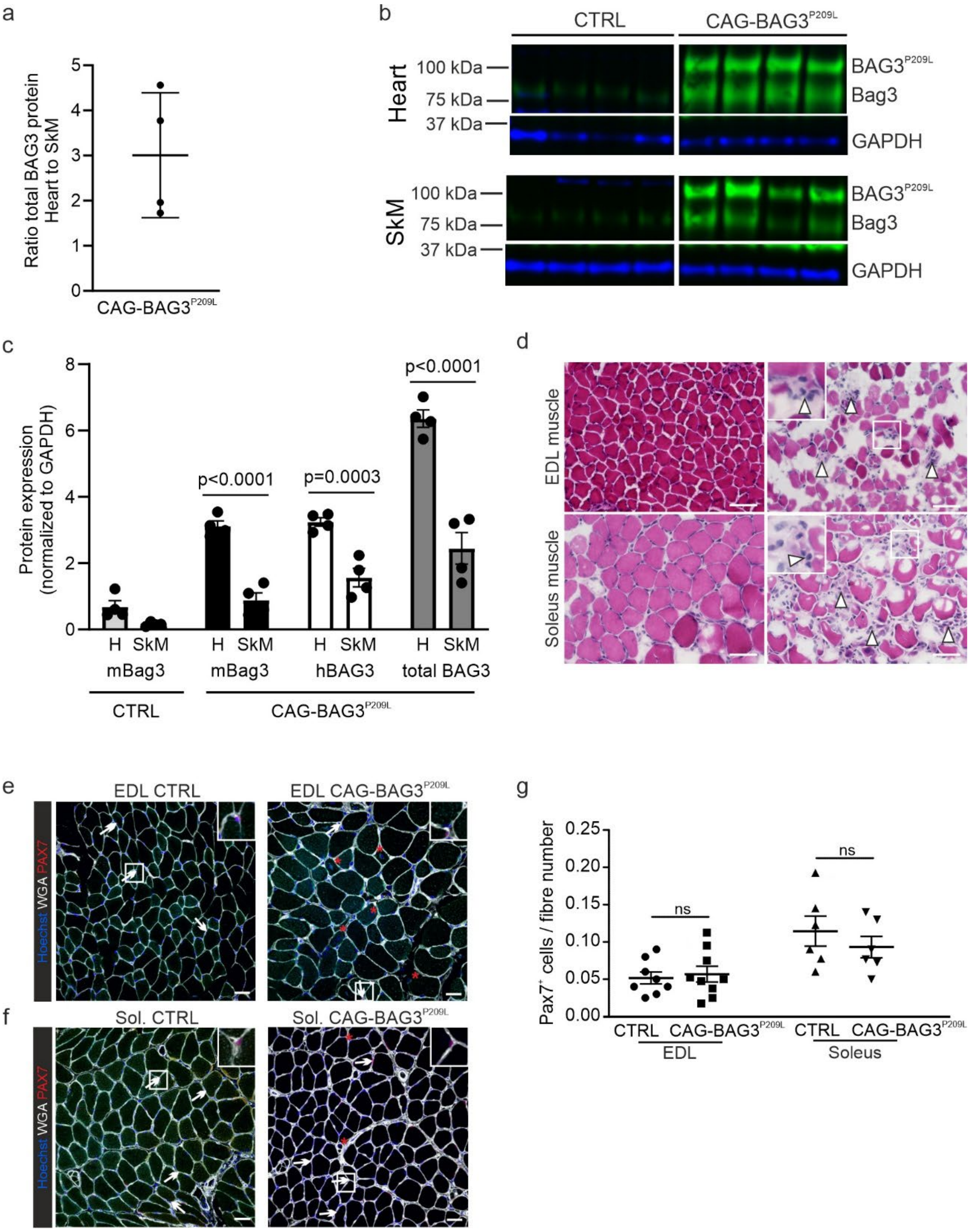

Supplementary Figure 3. Expression of BAG3 in control and CAG-BAG3-EGFP transgenic models.

(a) Quantification of total BAG3 protein showing the ratio of heart to skeletal muscle levels in CAG-BAG3^{P209L}-mice (n = 4 biologically independent mice per group). Data are shown as mean ± SEM. (b) Representative immunoblots of BAG3 protein expression in heart (H) and skeletal muscle (SkM) tissue from CTRL and CAG-BAG3^{P209L} mice (n = 4 biologically independent mice per group). GAPDH was used as loading control. (c) Densitometric quantification of protein expression levels of endogenous mBag3, exogenous hBAG3, and total BAG3 normalized to GAPDH in heart (H) and skeletal muscle (SkM) (n = 4 biologically independent mice per group). Shown is mean ± SEM, statistical test = two-

way repeated-measures ANOVA, followed by Šídák's multiple-comparisons test. (d) Hematoxylin-eosin staining demonstrated centralization of skeletal muscle cell nuclei of M. soleus and EDL from CAG-BAG3^{P209L} and CTRL-mice. In addition, infiltrating cells could be visualized (arrowheads, insets). (e,f) Cross-sections of M. soleus and the EDL stained with Pax7 and WGA showed red-stained satellite cells (arrows) between basal lamina and sarcolemma, as well as blue muscle cell nuclei, which are physiologically found at the edge of the muscle fiber but were sometimes in a centralized position in the muscles of homozygous mice (e,f, asteriks). (d-f) The experiments were repeated three times using biologically independent replicates, yielding similar results. (g) The number of satellite cells (Pax7^{+} cells/fiber count) did not differ significantly in either M. soleus (n = 6 biologically independent mice per group) or EDL (n = 9 biologically independent CAG-BAG3^{P209L}-mice, n = 8 biologically independent CTRL-mice). Mean ± SEM. Two-sided unpaired Student's t-test. Scale bars = 50 µm. Source data are provided as a Source Data file.

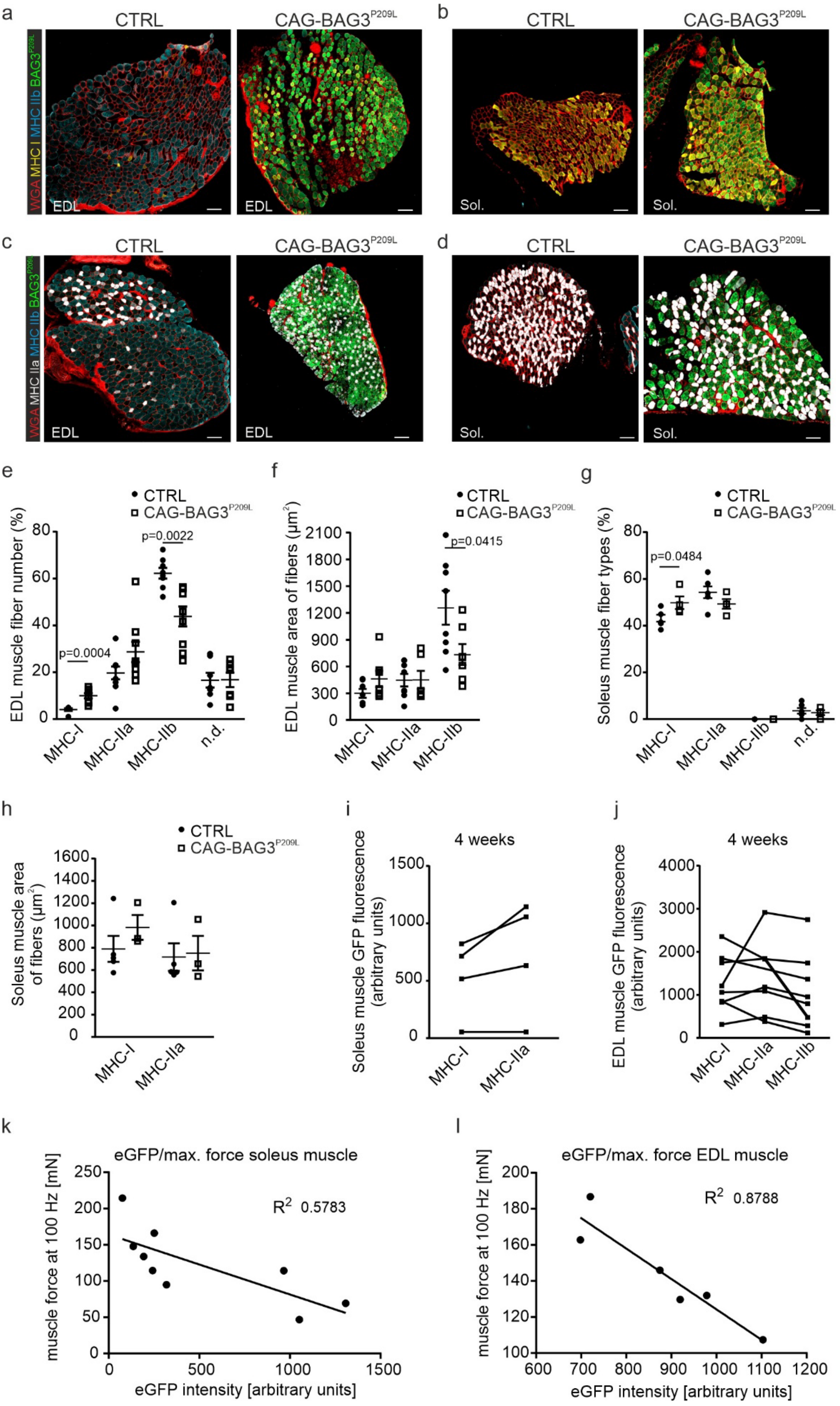

Supplementary Figure 4. Fiber type shift and loss of force in muscles from CAG-BAG^{P209L}-mice
(a,b) Staining against WGA (red), BA-F8 (MHC I, yellow), and BF-F8 (MHC IIb, blue) and (c,d) against WGA (red), SC-71 (MHC IIa, white), and BF-F8 (MHC IIb, blue) of soleus muscle and EDL of controls and CAG-BAG^{P209L} mice at 4 weeks of age. The EDL of the CAG-BAG^{P209L} mice had statistically more

type I fibers and fewer type IIb fibers (CAG-BAG3^{P209L}-mice: MHC-I n = 8/ MHC-IIa n = 9/ MHC-IIb n = 9/ n.d. n= 7; CTRL-mice: MHC-I n=6/ MHC-IIa n = 9/ MHC-IIb n = 8/ n.d. n= 9) (e). The soleus muscle of the CAG-BAG^{P209L} mice had statistically significantly more type-I fibers (CAG-BAG3^{P209L}-mice: MHC-I n = 7/ MHC-IIa n = 6/ MHC-IIb n = 7; CTRL-mice: MHC-I n=7/ MHC-IIa n = 8/ MHC-IIb n = 8) (f). The type-IIb fibers of the CAG-BAG^{P209L} mice were statistically significantly smaller (CAG-BAG3^{P209L}-mice: n = 4; CTRL-mice: n=6) (g). The size of the different fiber types was not significantly different (CAG-BAG3^{P209L}-mice: n = 3; CTRL-mice: n=5) (h). (e-h) shown is mean ± SEM, statistical test = two-sided unpaired Student's t-test. (a-h) All experiments were repeated three times using biologically independent replicates, yielding similar results; n denotes the number of biologically independent mice. (i,j) Quantification of maximal hBAG3–eGFP fluorescence intensity in individual muscle fibers of the soleus (i; n=4 biologically independent mice) and EDL (j; n=8 biologically independent mice) muscle from 4-week-old mice. No correlation was observed between hBAG3–eGFP expression levels and muscle fiber type in either muscle at this age. (k,l) Linear regression showed a significant linear relationship between force development in mN of the soleus muscle and the EDL when stimulated at 100 Hz and CAG-BAG3^{P209L}-eGFP expression, with a coefficient of determination of R2 0.5783 for the soleus muscle at 4 weeks (n = 9 biologically independent mice) (k) and R2 0.8788 for the EDL muscle at 4 weeks (n = 6 biologically independent mice) (l). Scale bars in a-d = 100 µm. Source data are provided as a Source Data file.

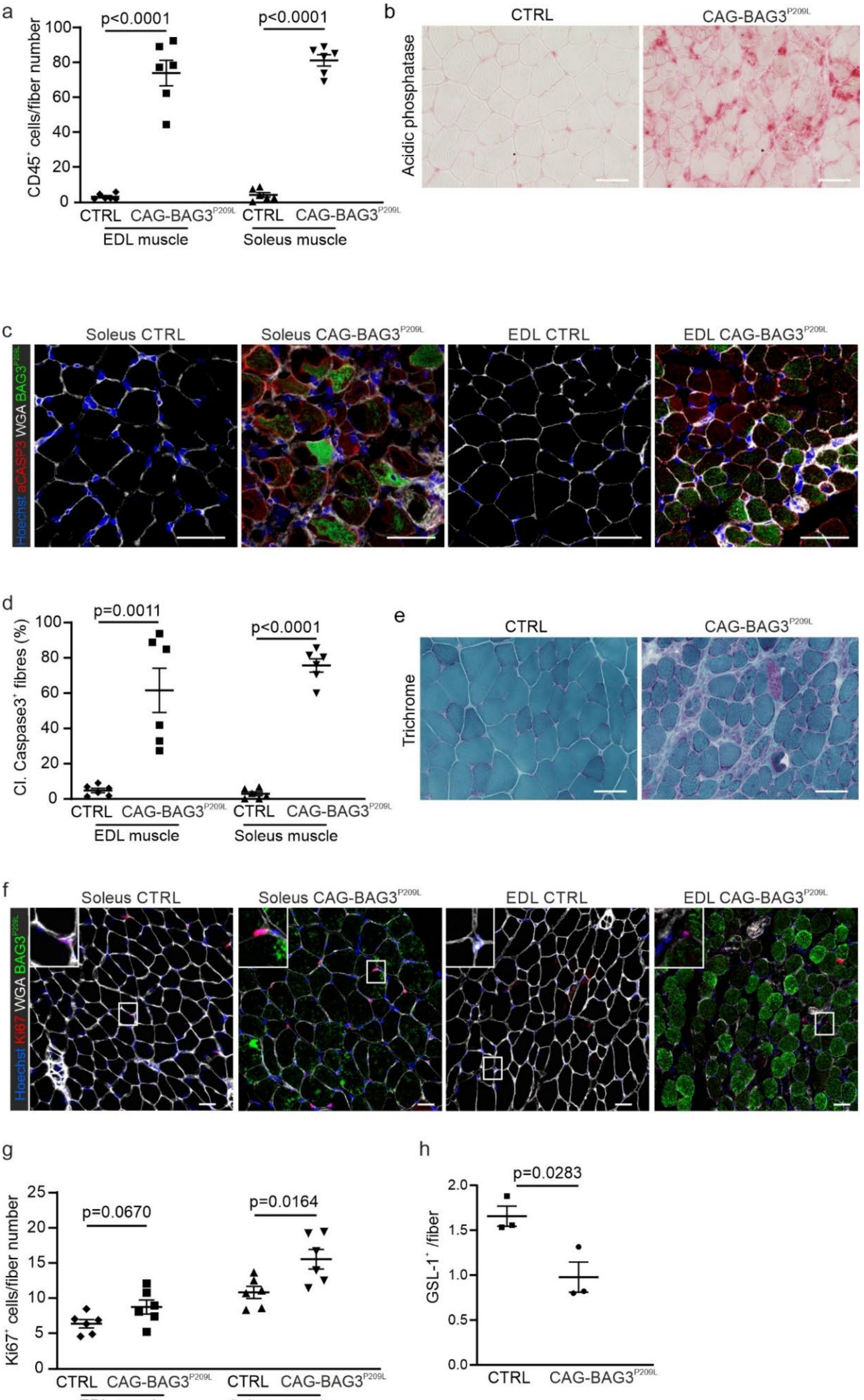

Supplementary Figure 5. Inflammatory cells and apoptotic fibers accumulate in skeletal muscle of CAG-BAG3^{P209L}-mice

(a) Quantification of CD45 positive cells per fiber in EDL and soleus muscle from CAG-BAG3^{P209L} and control mice (n = 4 biologically independent mice per group and per muscle). Shown is mean ± SEM, statistical test = two-sided unpaired Student's t-test. (b) Staining for acidic phosphatase (red) of quadriceps muscle from 4 weeks old CAG-BAG3^{P209L} and control mice. (c) Immunofluorescence staining of EDL and M. soleus from controls and from CAG-BAG3^{P209L}-mice at 4 weeks of age against cleaved caspase 3 (red). (d) Quantification revealed significantly more apoptotic skeletal muscle fibers in the EDL and soleus muscle of CAG-BAG3^{P209L}-mice than in controls (n = 6 biologically independent mice per group and per muscle). Shown is mean ± SEM, statistical test = two-sided unpaired Student's t-test. (e) Trichrome staining, showing fibrosis in CAG-BAG3^{P209L}-mice. (f) Immunofluorescence staining of EDL and M. soleus from controls and from CAG-BAG3^{P209L}-mice at 4 weeks of age against cleaved Ki67 (red), WGA, Hoechst nuclear dye (blue). (g) Quantification of Ki67 staining revealed an increase in the number of Ki67^{+} cells in soleus muscles of CAG-BAG3^{P209L}-mice (n = 6 biologically independent mice per group and per muscle). Shown is mean ± SEM, statistical test = two-sided unpaired Student's t-test. (h) Quantification of epithelial cells (GSL-1 staining) per muscle fiber revealed decrease in capillary density in quadriceps muscle from CAG-BAG3^{P209L}-mice. (n = 3 biologically independent mice). Shown is mean ± SEM, statistical test = two-sided unpaired Student's t-test. Scale bars = 50 µm (b,e), and 25 µm (c,f). (b,c,e,f) The experiments were repeated three times using biologically independent replicates, yielding similar results. Source data are provided as a Source Data file.

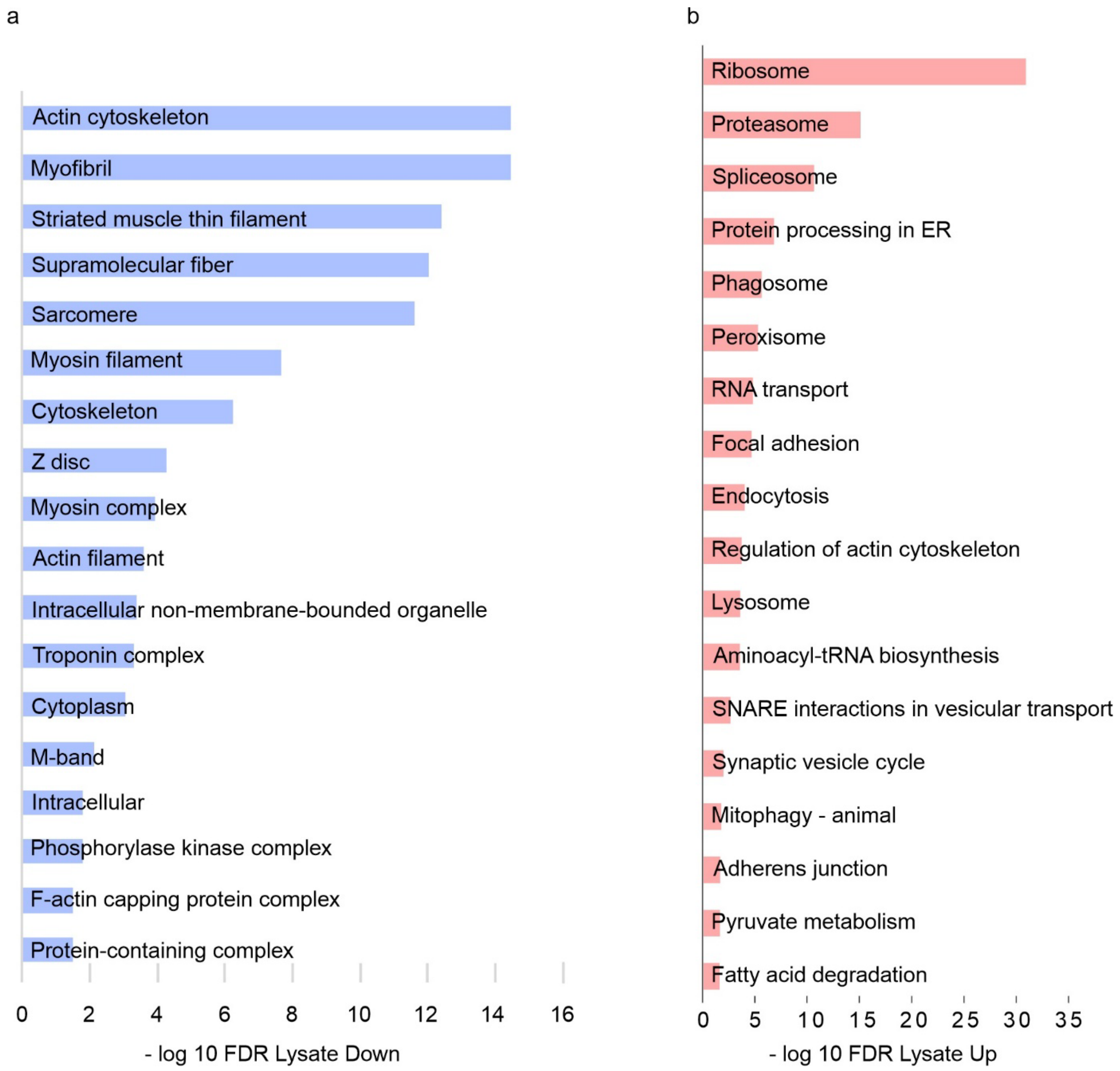

Supplementary Figure 6. Classification of proteins with differential abundance in CAG-BAG3^{P209L} mice compared to control.
(a) KEGG term enrichment for proteins accumulating in the total lysate and (b) GO-CC term enrichment for proteins depleted in the total lysate with an FDR corrected p-value ≤ 0.05. Source data are provided as a Source Data file.

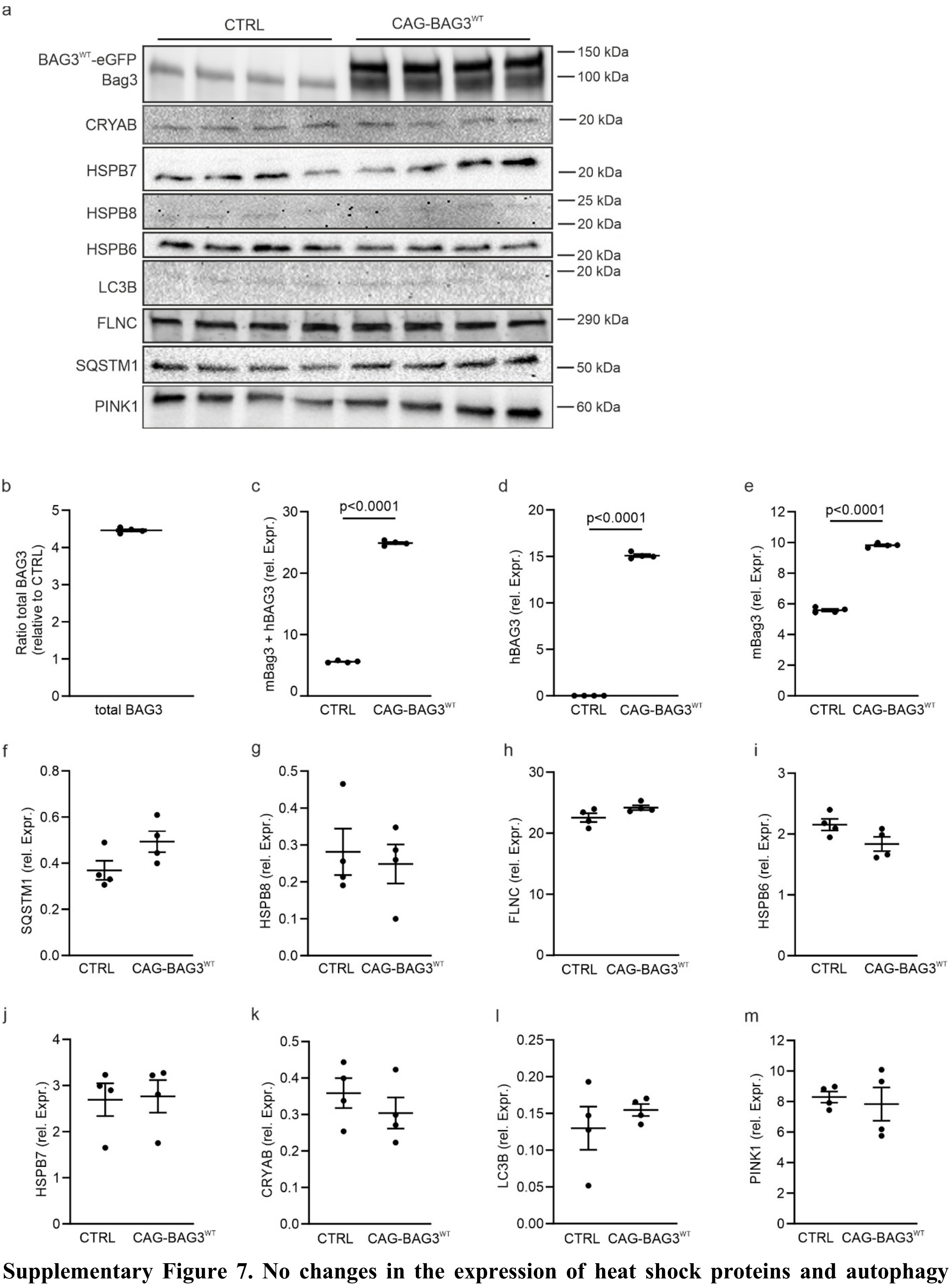

Supplementary Figure 7. No changes in the expression of heat shock proteins and autophagy markers in skeletal muscle from CAG-BAG3^{WT}-mice

(a) Western blot analyses were performed on M. quadriceps femoris of controls (CTRL) and CAG-BAG3^{WT}-mice at 4 weeks of age. (b-m) Quantification of the ratio of total BAG3 in CAG-BAG3^{WT}-mice to CTRL-mice (b), mouse Bag3 and human BAG3^{WT} (c), human BAG3^{WT} (d), mouse Bag3 (e), SQSTM1 (f), HSPB8 (g), FLNC (h), HSPB6 (i), HSPB7 (j), heat shock protein CRYAB (k), autophagy marker LC3B (l), and the mitophagy marker PINK1 (m) in skeletal muscle of CAG-BAG3^{WT}-mice. (b-

m) n represents the number of biologically independent mice, with four mice per group. Shown is the mean ± SEM, statistical test = two-sided unpaired Student's t-test, rel. Expr. = Expression relative to total protein. Source data are provided as a Source Data file.

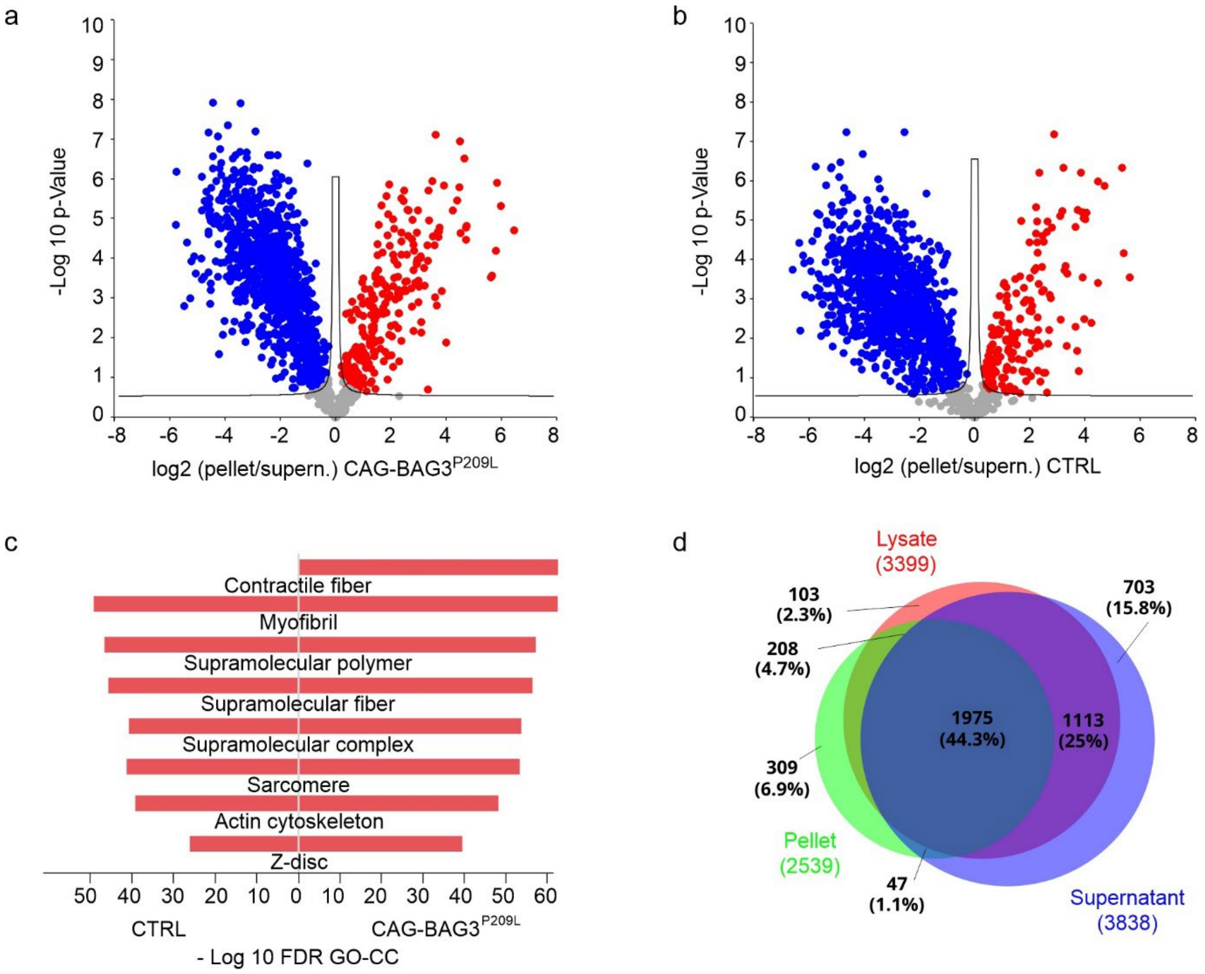

Supplementary Figure 8. Comparison of pellet and supernatant fractions in the control and the CAG-BAG3^{P209L}-mice samples.

Volcano plots comparing proteins accumulating in the pellet fraction compared to supernatant in quadriceps muscle from (a) CAG-BAG3^{P209L}-mice and (b) control mice. Proteins significantly changing with an FDR corrected p-value ≤ 0.05 are highlighted in red (accumulating in the pellet) and blue (depleted pellet). (c) Functional classification by GO-CC term enrichment for proteins accumulating in the pellet of control and CAG-BAG3^{P209L}-mice is shown here. Enrichment of similar terms in the control and the CAG-BAG3^{P209L}-mice shows that the fractionation procedure enriched the cytoskeletal-associated proteins as intended. (d) Venn-diagram depicting the overlap of proteins identified in the pellet, supernatant, and the total lysate of the mouse muscle. Source data are provided as a Source Data file.

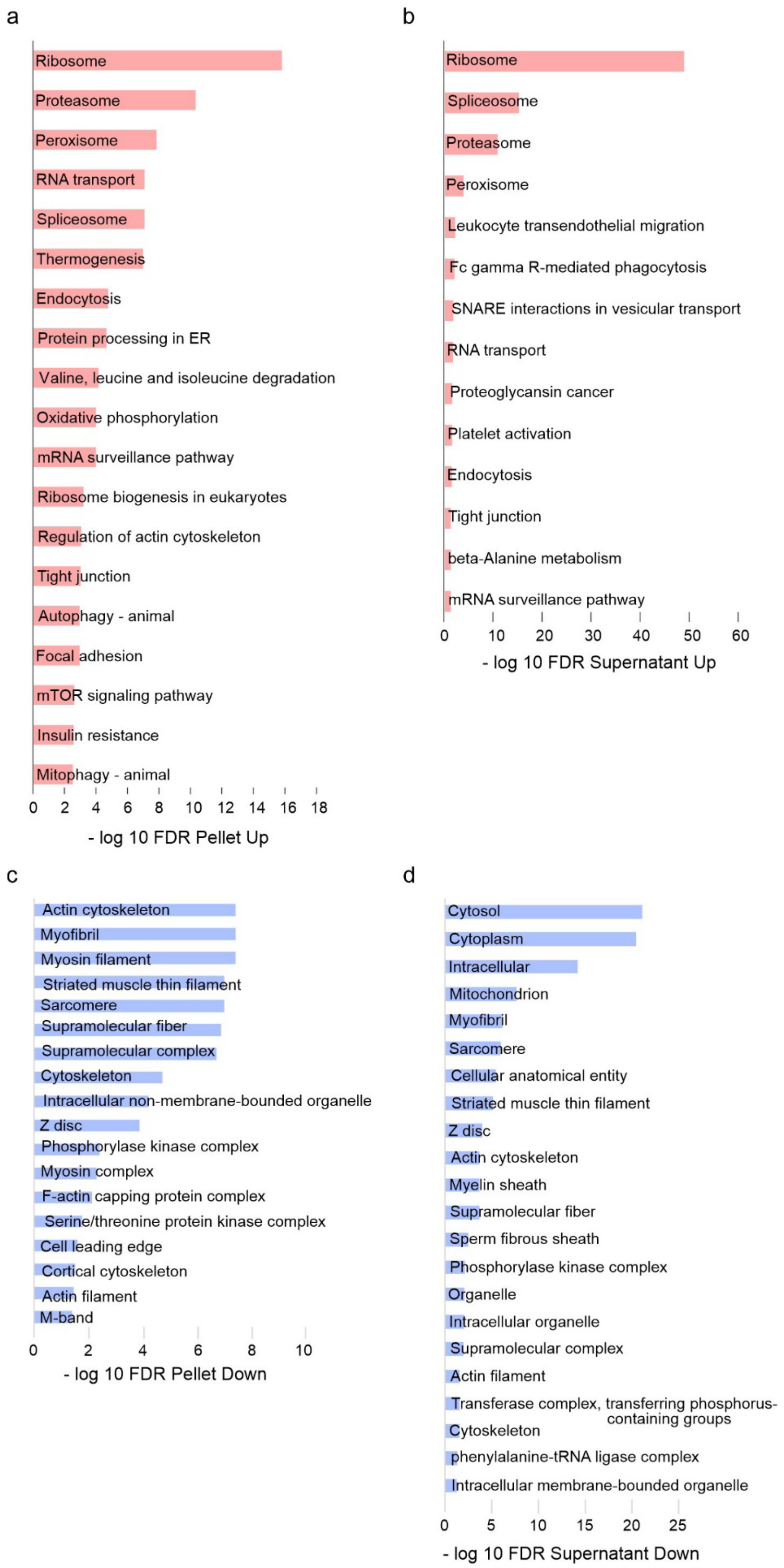

Supplementary Figure 9. Quantitative analysis of fractionated skeletal muscle.

(a-d) KEGG term enrichment for proteins accumulating and GO-CC term enrichment for proteins depleted in the pellet and the supernatant fraction from quadriceps muscle from CAG-BAG3^{P209L}-mice. Only selected terms with an FDR-corrected p-value ≤ 0.05 are shown here. A complete list is available in the supplementary table. Source data are provided as a Source Data file.

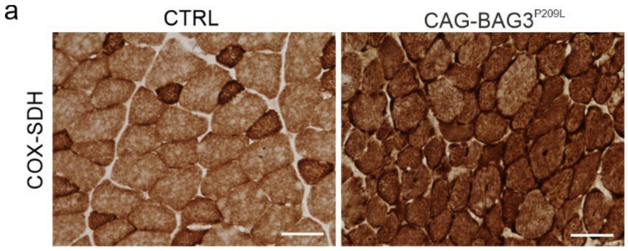

Supplementary Figure 10. Accumulation of mitochondria in muscle from CAG-BAG3^{P209L}-mice
(a) Staining of sections from quadriceps muscle from control and CAG-BAG3^{P209L}-mice for COX-SDH, showing increased COX activity due to mitochondria accumulation. Scale bar = 50 µm. Source data are provided as a Source Data file.

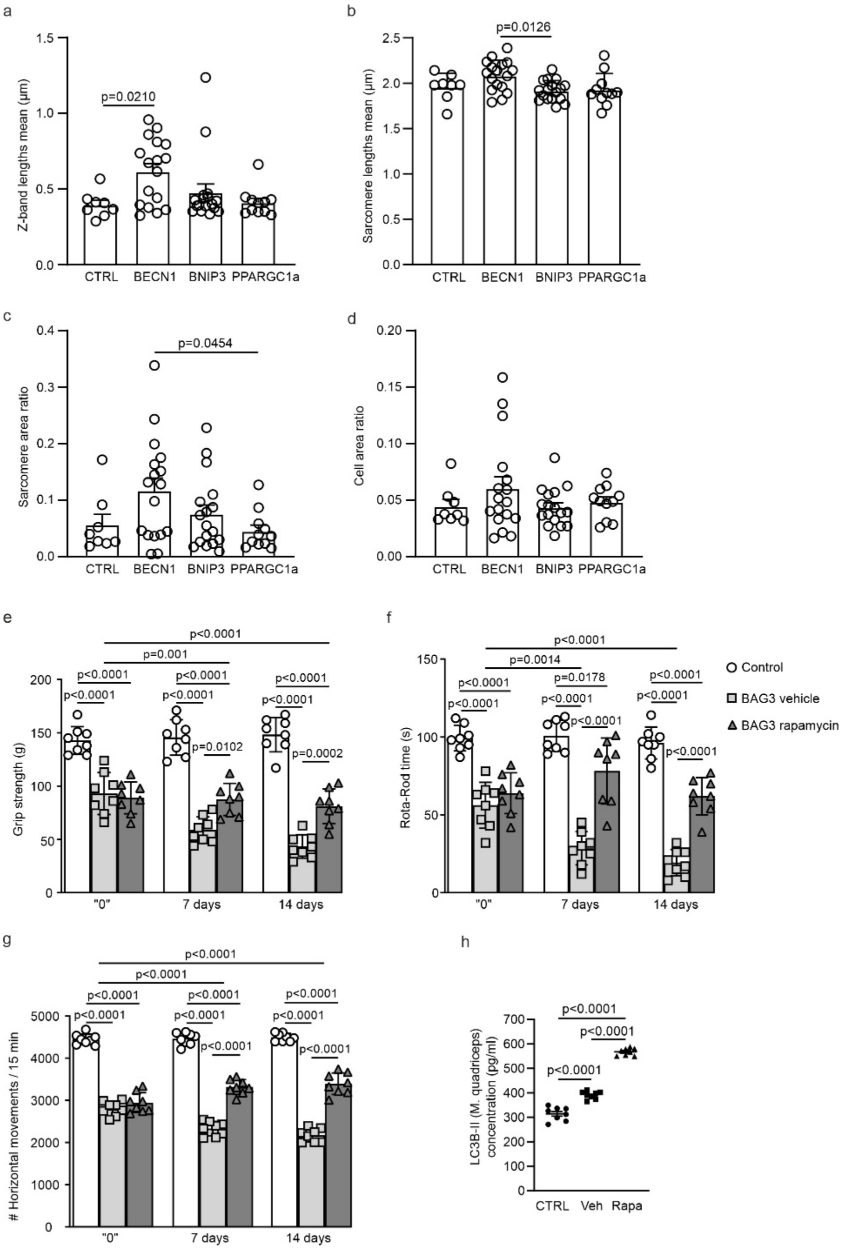

Supplementary Figure 11. Phenotype in skeletal muscle of CAG-BAG^{P209L} mice is due to a blockade of autophagy

(a-d) Quantification of Z-band lengths mean (a), sarcomere lengths mean (b), sarcomere area ratio (c), and cell area ratio (d) by the SarcAsM algorithm (n denotes the number of biologically independent mice: CTRL n=8/ BECN1 n=17/ BNIP3 n=17/ PPARGC1A n=11). One-way ANOVA with Dunnett's multiple comparisons test.

(e-g) Grip strength (e) and Rota-Rod performance (f) and voluntary movements (g) were measured in non-transgenic untreated mice (CTRL) and CAG-BG3^{P209L} mice treated with vehicle (Veh) or rapamycin (Rapa, daily i.p. injection of 8 mg/kg rapamycin for 14 days) at 0, 7, and 14 days post-treatment. Rapamycin improved motor function in CAG-BG3^{P209L} mice significantly over time. Data are presented as mean ± SD; n = 8 biologically independent mice per group. Two-way repeated-measures ANOVA with Bonferroni post hoc test. (h) Immunoblot analysis and quantification of LC3B-II expression in quadriceps muscle from CAG-BAG3^{P209L}-mice showed an increase after 14 days of treatment with rapamycin compared to untreated non-transgenic mice or vehicle controls, indicating induction of autophagy. Data are presented as mean ± SD; n = 8 biologically independent mice per group. One-way ANOVA with Bonferroni post hoc test. Source data are provided as a Source Data file.

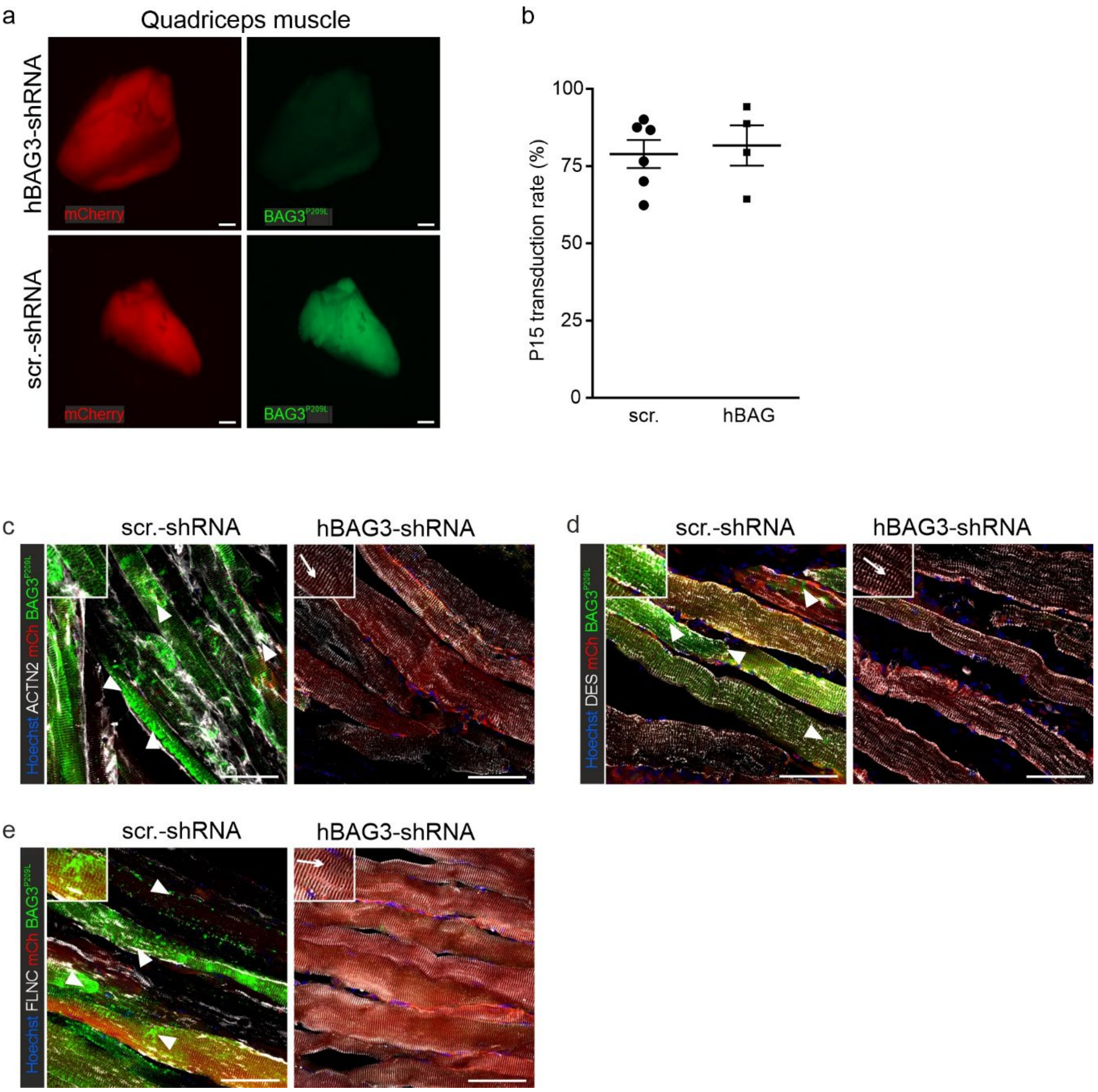

Supplementary Figure 12. shRNA-mediated knockdown of hBAG3^{P209L} *in vivo*

(a) Overview of quadriceps muscles from AAV/rh10 treated CAG-BAG3^{P209L} mice at P37. hBAG3^{P209L}-eGFP expression was reduced after treatment with AAV/rh10 hBAG3 shRNA. Scale bars: 500 µm. (b) Quantification of the transduction rate by mCherry expression as percentage of total skeletal myocytes (n = 6 biologically independent CAG-BAG3^{P209L} mice with AAV/rh10 scrambled shRNA; n = 4 biologically independent CAG-BAG3^{P209L} mice with AAV/rh10 BAG3 shRNA). Mean ± SEM. Statistical test = two-sided unpaired Student's t-test. (c-e) Sections of quadriceps muscle from 5 weeks old CAG-BAG3^{P209L}-mice which were treated with either AAV/rh10 BAG3 shRNA or AAV/rh10 scrambled shRNA were stained for ACTN2 (c), DES (d), FLNC (e). Arrowheads depict BAG3^{P209L}-eGFP-containing protein aggregates; arrows depict intact sarcomere structures. Scale bars: 50 µm. (a,c-e) All experiments were repeated three times using biologically independent replicates, yielding similar results. Source data are provided as a Source Data file.

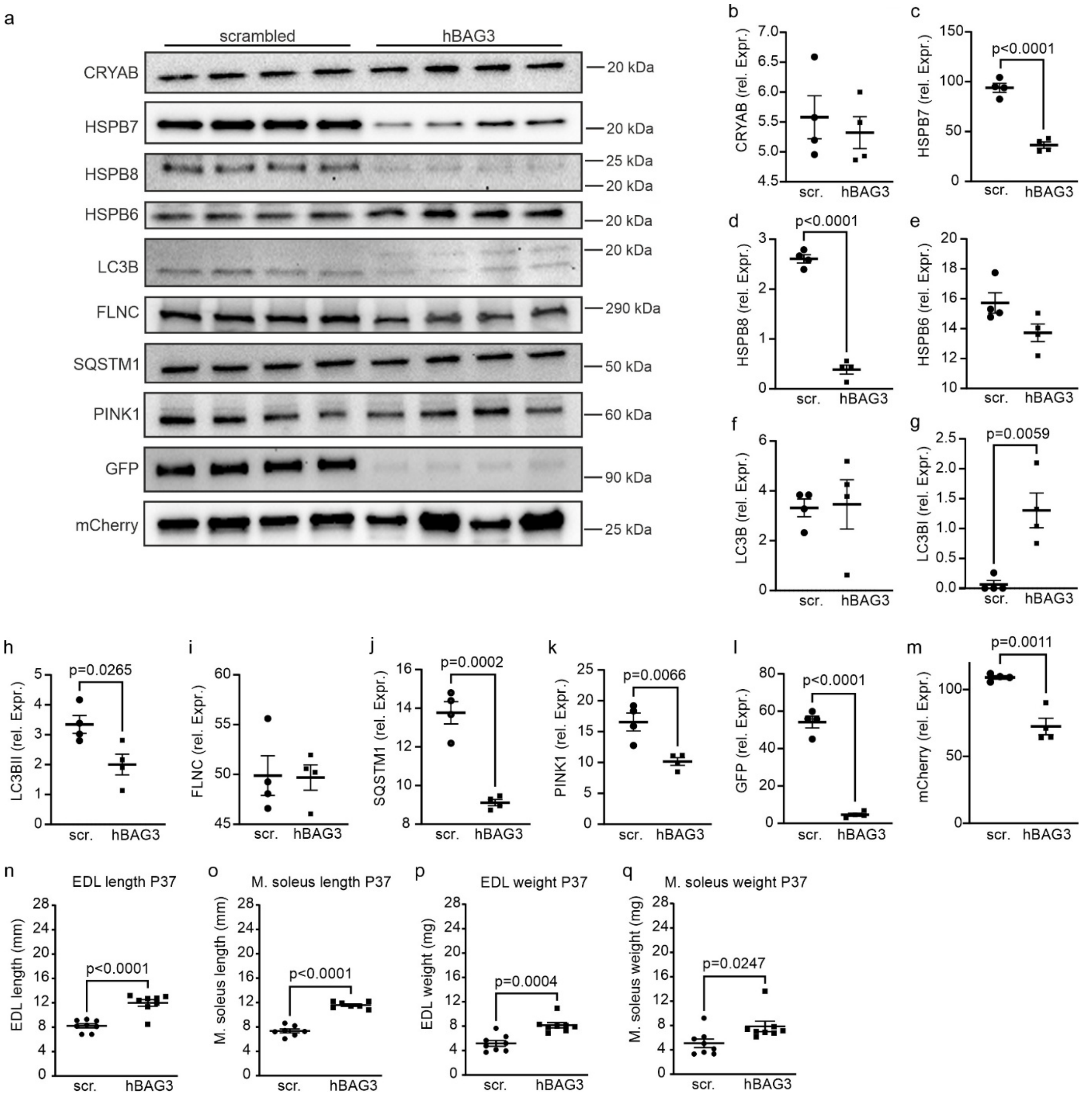

Supplementary Figure 13. Western blot analyses of quadriceps femoris muscle of CAG-BAG3^{P209L}-mice after shRNA-mediated knockdown of hBAG3^{P209L} *in vivo*

(a) Western blot analyses of M. quadriceps femoris of CAG-BAG3^{P209L}-mice treated with AAV/rh10 BAG3 shRNA or AAV/rh10 scramble shRNA. (b-m) Quantification of CRYAB (b), HSPB7 (c), HSPB8 (d), HSPB6 (e), LC3B (f), LC3B-I (g), LC3B-II (h), FLNC (i), SQSTM1 (j), PINK1 (k), GFP (l), and mCherry (m) in skeletal muscle of CAG-BAG3^{P209L}-mice treated with AAV/rh10 BAG3 shRNA or AAV/rh10 scramble shRNA. (b-m) n represents the number of biologically independent mice, with four mice per group. Shown is the mean ± SEM, statistical test = two-sided unpaired Student's t-test, rel. Expr. = Expression relative to total protein. Length (o,p) (Soleus: n= 7 biologically independent mice per group; EDL: n= 8 biologically independent mice per group) and weight (q,r) (n= 8 biologically independent mice per group per muscle) of EDL and soleus muscles were significantly increased after treatment with AAV/rh10 BAG3 shRNA when compared to AAV/rh10 scrambled shRNA. Shown is the mean ± SEM, statistical test = two-sided unpaired Student's t-test. Source data are provided as a Source Data file.

```
Human BAG3 alligned to mouse Bag3

Alignment statistics for match #1 Score Expect Method Identities   Positives   Gaps
836 bits(2160) 0.0  Compositional matrix adjust. 484/581(83%)    508/581(87%)
10/581(1%)

Human  1    MSAATHSPMMQVASGNG--DRDPLPPGWEIKIDPQTGWPFFVDHNSRTTTWNDPRVPSEG  58
            MSAAT SPMMQ+ASGNG  DRDPLPPGWEIKIDPQTGWPFFVDHNSRTTTWNDPRVP EG
Mouse  1    MSAATQSPMMQMASGNGASDRDPLPPGWEIKIDPQTGWPFFVDHNSRTTTWNDPRVPPEG  60

Human  59   PKETPSSANGPSREGSRLPPAREGHPVYPQLRPGYIPIPVLHEGAENRQVHPFHVYPQPG  118
            PK+T SSANGPSR+GSRL P REGHP+YPQLRPGYIPIPVLHEG+ENRQ H FH Y QPG
Mouse  61   PKDTASSANGPSRDGSRLLPIREGHPIYPQLRPGYIPIPVLHEGSENRQPHLFHAYSQPG  120

Human  119  MQRFRTEAAAAAPQRSQSPLRG-MPETTQPDKQCGQVAAAAAAQPPAS---HGPERSQSP  174
            +QRFRTEAAAA PQRSQSPLRG M E  Q DKQCGQ+ A A          HGPERSQSP
Mouse  121  VQRFRTEAAAATPQRSQSPLRGGMTEAAQTDKQCGQMPATATTAAAQPPTAHGPERSQSP  180

Human  175  AASDCSSSSSSASLPSSGRSSLGSHQLPRGYISIPVIHEQNVTRPAAQPSFHQAQKTHYP  234
            AASDCSSSSSSASLPSSGRSSLGSHQLPRGYI IPVIHEQN+TRPAAQPSFHQAQKTHYP
Mouse  181  AASDCSSSSSSASLPSSGRSSLGSHQLPRGYIPIPVIHEQNITRPAAQPSFHQAQKTHYP  240

Human  235  AQQGEYQTHQPVYHKIQGDDWEPRPLRAASPFRSSVQGASSREGSPARSSTPLHSPSPIR  294
            AQQGEYQ  QPVYHKIQGDDWEPRPLRAASPFRS V+GASSREGSPARS TP+H PSPIR
Mouse  241  AQQGEYQPQQPVYHKIQGDDWEPRPLRAASPFRSPVRGASSREGSPARSGTPVHCPSPIR  300

Human  295  VHTVVDRPQQPMTHRETAPVSQPENKPESKPGPVGPELPPGHIPIQVIRKEVDSKPVSQK  354
            VHTVVDRPQ PMTHRE  PV+QPENKPESKPGP GP+LPPGHIPIQVIR+E DSKPVSQK
Mouse  301  VHTVVDRPQ-PMTHREPPPVTQPENKPESKPGPAGPDLPPGHIPIQVIRREADSKPVSQK  359

Human  355  PPPPSEKVEVKVPPAPVPCPPPSPGPSAVPSSPKSVATEERAAPSTAPAEATPPKPGEAE  414
             PPP+EKVEVKV  AP+PCP PSP PSAVPS PK+VA E++AAPS APAE   PK GEAE
Mouse  360  SPPPAEKVEVKVSSAPIPCPSPSPAPSAVPSPPKNVAAEQKAAPSPAPAEPAAPKSGEAE  419

Human  415  APPKHPGVLKVEAILEKVQGLEQAVDNFEGKKTDKKYLMIEEYLTKELLALDSVDPEGRA  474
             PPKHPGVLKVEAILEKVQGLEQAVD+FEGKKTDKKYLMIEEYLTKELLALDSVDPEGRA
Mouse  420  TPPKHPGVLKVEAILEKVQGLEQAVDSFEGKKTDKKYLMIEEYLTKELLALDSVDPEGRA  479

Human  475  DVRQARRDGVRKVQTILEKLEQKAIDVPGQVQVYELQPSNLEADQPLQAIMEMGAVAADK  534
            DVRQARRDGVRKVQTILEKLEQKAIDVPGQVQVYELQPSNLEA+QPLQ I  MGAV ADK
Mouse  480  DVRQARRDGVRKVQTILEKLEQKAIDVPGQVQVYELQPSNLEAEQPLQEI--MGAVVADK  537

Human  535  GKKNAGNAEDPHTETQQPEATAAATSNPSSMTDTPGNPAAP  575
             KK   N +DP TE+QQ EA AA   NPS+  D+ GN  AP
Mouse  538  DKKGPEN-KDPQTESQQLEAKAATPPNPSNPADSAGNLVAP  577
```

Supplementary Figure 14. Alignment of human BAG3 to mouse Bag3

Supplementary Table 1: Primers used for genotyping of transgenic mice

**Name**	**Sequence**	**Purpose**
oIMR 9021 common rev	5´-CCGAAAATCTGTGGGAAGTC-3´	Wildtype (Rosa26 Locus): Band (297 bp) = P209L hemizygous / no Band = P209L homozygous
oIMR 9020 wt fw	5´-AAGGGAGCTGCAGTGGAGTA-3´	Wildtype (Rosa26 Locus): Band (297 bp) = P209L hemizygous / no Band = P209L homozygous
CAG2-fw	5´-TTCGGCTTCTGGCGTGTGAC-3´	Genotyping of Tg(CAG-flox-hBAG3^{WT}-eGFP) and Tg(CAG-flox-hBAG3^{P209L}-eGFP) transgenic mice

		515 bp if floxed cassette excised vs. 3 kb if not
BAG3-h-spez.tev	5´-GCACAGGAATGGGAATGTAG-3´	Genotyping of Tg(CAG-flox-hBAG3^{WT}-eGFP) and Tg(CAG-flox-hBAG3^{P209L}-eGFP) transgenic mice<br>515 bp if floxed cassette excised vs. 3 kb if not
loxP-hygro-genotyp-BAG3 fw	TCC CTG CCA CAG TCT GAG AG	Genotyping of Tg(CAG-flox-hBAG3^{WT}-eGFP) and Tg(CAG-flox-hBAG3^{P209L}-eGFP) transgenic mice<br>To test whether the floxed cassette was cut out (no band (cut) vs 463 bp)
BAG3 human-spez rev1	AGC GCC TTC ATG GAG CAC AG	Genotyping of Tg(CAG-flox-hBAG3^{WT}-eGFP) and Tg(CAG-flox-hBAG3^{P209L}-eGFP) transgenic mice<br>To test whether the floxed cassette was cut out (no band (cut) vs 463 bp)

Original Blots for Suppl. Fig. 3c

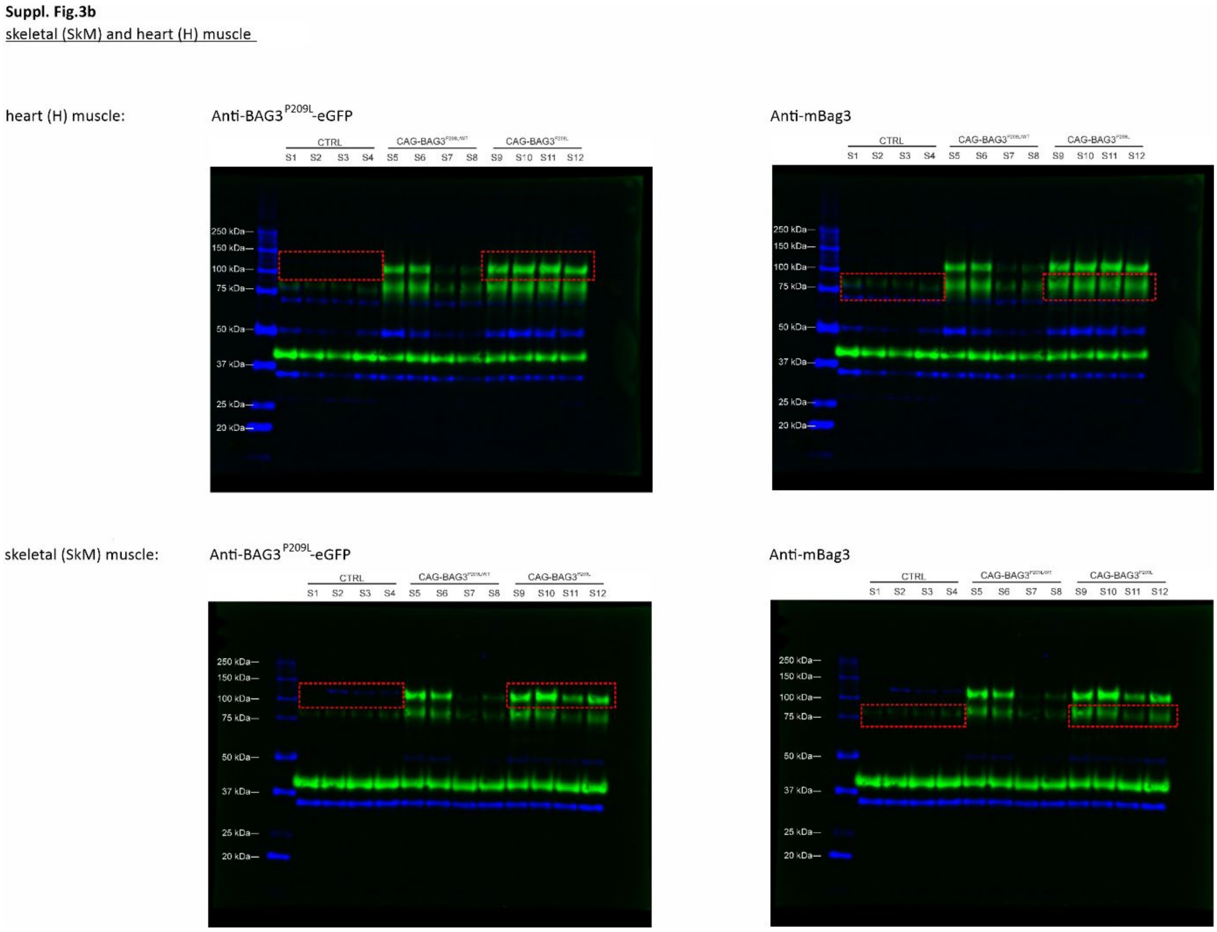

Original Blots for Suppl. Fig. 7a

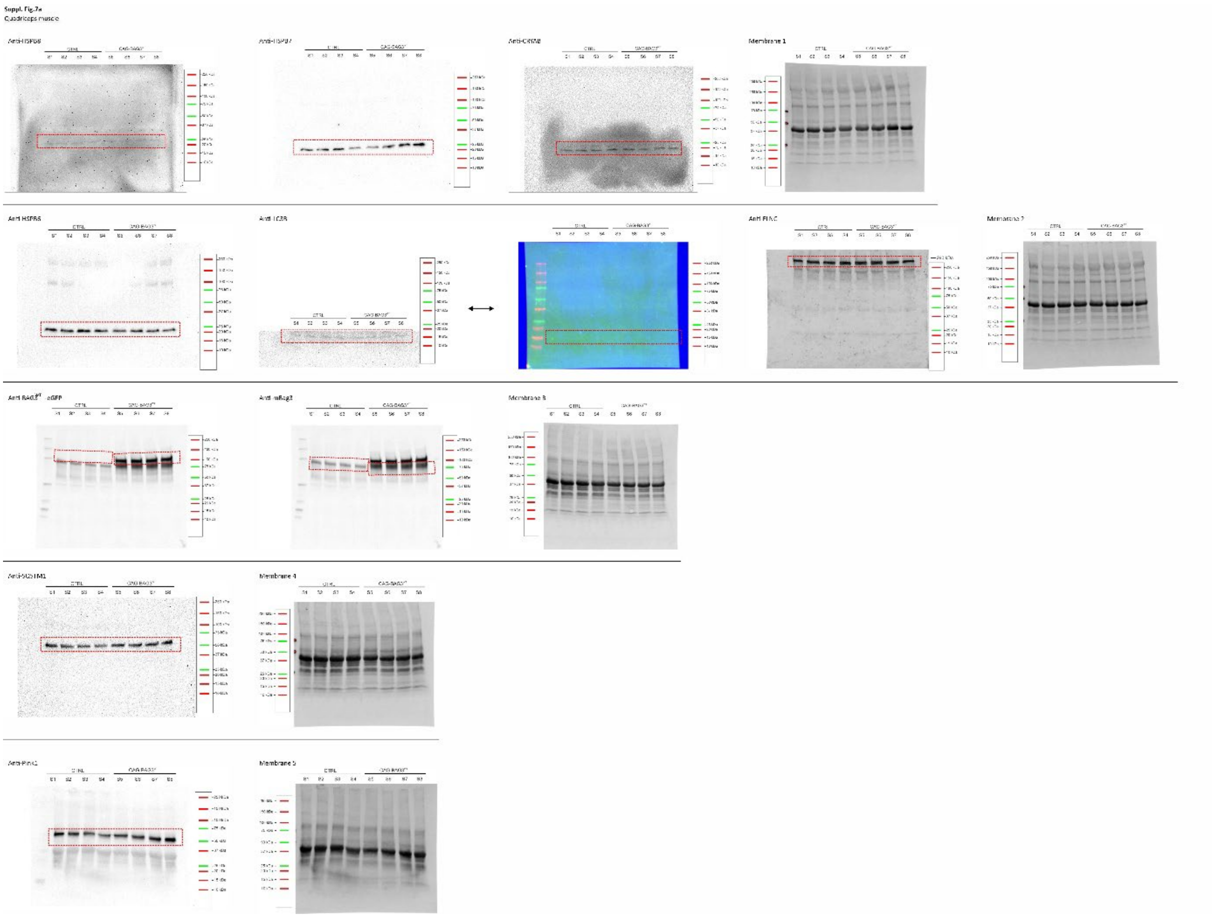

Original Blots for Suppl. Fig. 13a

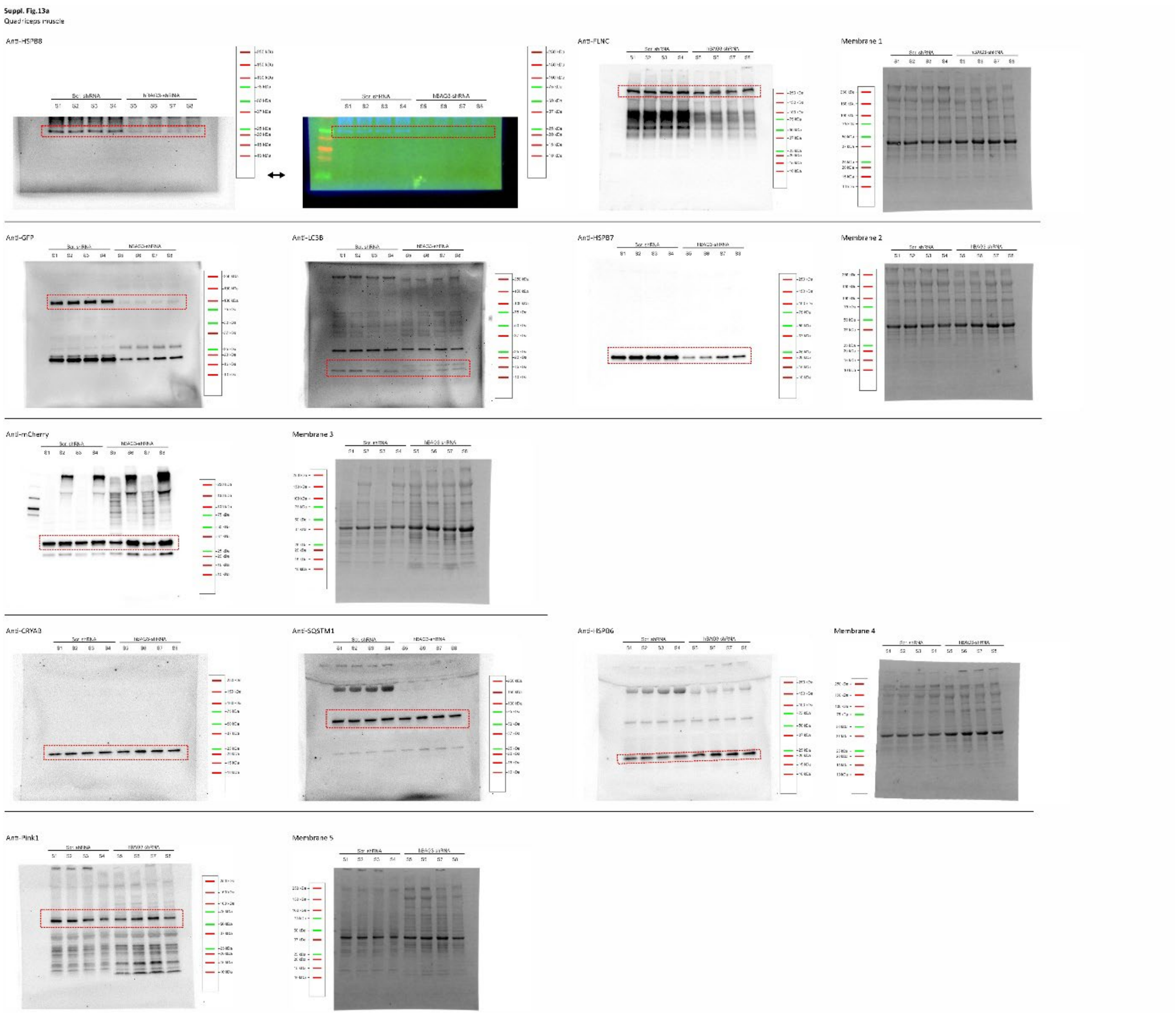

Supplement: Supplementary file 1 — Supplementary Information [file 41467_2026_71749_MOESM1_ESM.pdf]
