# Supplementary material for: Blockage of autophagy causes severe skeletal muscle disruption in a mouse model for myofibrillar myopathy 6
Source: Nat Commun. 2026 Apr 11;17:3436. doi: 10.1038/s41467-026-71749-6 (PMC13076893; doi:10.1038/s41467-026-71749-6)
Supplement: Supplementary file 2 — Description of Additional Supplementary Files [file 41467_2026_71749_MOESM2_ESM.pdf]

Description of Additional Supplementary Files

File name: Supplementary Data 1.
Description: Table S1_RNA-Seq_volcano plot
Differentially expressed genes from tissue RNA-seq analysis as determined by DeSeq2 (Two-sided Wald test statistic). This table was used to create the volcano plot in Fig. 4a. Base mean: mean normalised counts, averaged over all samples from both conditions. log2(FC): the logarithm (to basis 2) of the fold change (See the note in inputs section). StdErr: standard error estimate for the log2 fold change estimate. Wald-Stats: Wald statistic. P-value: p value for the statistical significance of this change. P-adj: p value adjusted for multiple testing with the Benjamini-Hochberg procedure which controls false discovery rate (FDR).

File name: Supplementary Data 2.
Description: Table S2_RNA-Seq_Heatmap
Table of all significantly differentially expressed genes from tissue RNA-seq analysis as determined by DeSeq2 (Two-sided Wald test statistic) with P-adj <0.05. This table was used to create the heatmap in Fig. 4b. Base mean: mean normalised counts, averaged over all samples from both conditions. log2(FC): the logarithm (to basis 2) of the fold change (See the note in inputs section). StdErr: standard error estimate for the log2 fold change estimate. Wald-Stats: Wald statistic. P-value: p value for the statistical significance of this change. P-adj: p value adjusted for multiple testing with the Benjamini-Hochberg procedure which controls false discovery rate (FDR).

File name: Supplementary Data 3.
Description: Table S3_Lysate_sign_change
Quantitative proteomic analysis of proteins identified in the total lysate of control and CAG-BAG3^{P209L} mice. Only proteins quantified with unique peptides were included. Significantly differentially abundant proteins were identified using a two-tailed Student's t-test with FDR correction ($q \le 0.05$).

File name: Supplementary Data 4.
Description: Table S4_Lysate_unique
List of proteins identified uniquely in the control or the C CAG-BAG3^{P209L} mice in the total lysate sample.

File name: Supplementary Data 5.
Description: Table S5_pellet_sign_change
Quantitative proteomic analysis of proteins identified in the pellet fraction of control and CAG-BAG3^{P209L} mice. Only proteins quantified with unique peptides were included. Significantly differentially abundant proteins were identified using a two-tailed Student's t-test with FDR correction ($q \le 0.05$).

File name: Supplementary Data 6.
Description: Table S6_pellet_unique
List of proteins identified uniquely in the control or the CAG-BAG3^{P209L} mice in the pellet sample.

File name: Supplementary Data 7.
Description: Table S7_supernatant_sign_chang
Quantitative proteomic analysis of proteins identified in the supernatant fraction of control and CAG-BAG3^{P209L} mice. Only proteins quantified with unique peptides were included. Significantly differentially abundant proteins were identified using a two-tailed Student's t-test with FDR correction ($q \le 0.05$).

File name: Supplementary Data 8.
Description: Table S8_supernatant_unique
List of proteins identified uniquely in the control or the CAG-BAG3^{P209L} mice in the supernatant sample.
