# Supplementary material for: Blockage of autophagy causes severe skeletal muscle disruption in a mouse model for myofibrillar myopathy 6
Source: Nat Commun. 2026 Apr 11;17:3436. doi: 10.1038/s41467-026-71749-6 (PMC13076893; doi:10.1038/s41467-026-71749-6)
Supplement: Supplementary file 11 — Reporting Summary [file 41467_2026_71749_MOESM11_ESM.pdf]

nature portfolio

Corresponding author(s): Michael Hesse

Last updated by author(s): Feb 5, 2026

Reporting Summary

Nature Portfolio wishes to improve the reproducibility of the work that we publish. This form provides structure for consistency and transparency in reporting. For further information on Nature Portfolio policies, see our Editorial Policies and the Editorial Policy Checklist.

Statistics

For all statistical analyses, confirm that the following items are present in the figure legend, table legend, main text, or Methods section.

n/a	Confirmed	
☐	☒	The exact sample size (n) for each experimental group/condition, given as a discrete number and unit of measurement
☐	☒	A statement on whether measurements were taken from distinct samples or whether the same sample was measured repeatedly
☐	☒	The statistical test(s) used AND whether they are one- or two-sided<br>*Only common tests should be described solely by name; describe more complex techniques in the Methods section.*
☒	☐	A description of all covariates tested
☐	☒	A description of any assumptions or corrections, such as tests of normality and adjustment for multiple comparisons
☐	☒	A full description of the statistical parameters including central tendency (e.g. means) or other basic estimates (e.g. regression coefficient) AND variation (e.g. standard deviation) or associated estimates of uncertainty (e.g. confidence intervals)
☐	☒	For null hypothesis testing, the test statistic (e.g. F, t, r) with confidence intervals, effect sizes, degrees of freedom and P value noted<br>*Give P values as exact values whenever suitable.*
☒	☐	For Bayesian analysis, information on the choice of priors and Markov chain Monte Carlo settings
☒	☐	For hierarchical and complex designs, identification of the appropriate level for tests and full reporting of outcomes
☐	☒	Estimates of effect sizes (e.g. Cohen's d, Pearson's r), indicating how they were calculated

Our web collection on statistics for biologists contains articles on many of the points above.

Software and code

Policy information about availability of computer code

Data collection	HyStar Software v3.2 for proteomics data acquisition<br>ChemiDocTM MP imaging system with Image Lab v5.2.1 for immunoblot acquisition<br>Axiovision Rel. 4.8 Software for microscopical picture acquisition<br>LabChart Pro v7.3.7 ADInstruments for skeletal muscle force measurement

Data analysis	GraphPad Prism V9.0.1 for statistical analysis<br>PS Power and Sample Size Calculations programm V3.1.6 for calculation of sample size for animal experiments<br>BioRad's Image Lab 5.2.1 for quantification of immunoblots<br>Perseus version 1.6.10.0 for analysis of label-free quantification (LFQ) data<br>MaxQuant version 1.6.6.0 and 1.6.10.43 for analysis of proteomics data<br>Fiji (ImageJ) version 1.47n software package for quantification of protein gels<br>Galaxy platform version 2021 for analysis of tissue RNA-seq data<br>ClueGO v2.5.6 app in cytoscape v3.7.1 for gene ontology analysis<br>Axiovision Rel. 4.8 Software for picture analysis<br>DESeq2 version 2.11.40.6 for analysis of tissue RNA-seq data<br>RNA STAR version 2.7.2b for analysis of tissue RNA-seq data<br>featureCounts version 1.6.4+galaxy2 for analysis of tissue RNA-seq data<br>heatmap2 version 3.0.1 for analysis of tissue RNA-seq data<br>Volcano plot version 0.0.3 for analysis of tissue RNA-seq data<br>SarcAsM v0.2.0 to analyze sarcomere organization<br>LabChart v8.1.25 ADInstruments for analysis of skeletal muscle force

For manuscripts utilizing custom algorithms or software that are central to the research but not yet described in published literature, software must be made available to editors and reviewers. We strongly encourage code deposition in a community repository (e.g. GitHub). See the Nature Portfolio guidelines for submitting code & software for further information.

Data

Policy information about availability of data

All manuscripts must include a data availability statement. This statement should provide the following information, where applicable:

- Accession codes, unique identifiers, or web links for publicly available datasets
- A description of any restrictions on data availability
- For clinical datasets or third party data, please ensure that the statement adheres to our policy

The mass spectrometry proteomics raw data generated in this study have been deposited to the ProteomeXchange Consortium via the PRIDE29 partner repository under accession code PXD047942 [https://www.ebi.ac.uk/pride/archive/projects/PXD047942]. The tissue RNA-seq raw data used in this study are available in the SRA database under accession code PRJNA1082302 [https://www.ncbi.nlm.nih.gov/bioproject/PRJNA1082302]. The mass spectrometry proteomics data and the tissue RNA-seq data generated in this study are provided in the Supplementary Information/Source Data file.

Research involving human participants, their data, or biological material

Policy information about studies with human participants or human data. See also policy information about sex, gender (identity/presentation), and sexual orientation and race, ethnicity and racism.

Reporting on sex and gender	The study did not include research on human participants or human biological material.
Reporting on race, ethnicity, or other socially relevant groupings	*Please specify the socially constructed or socially relevant categorization variable(s) used in your manuscript and explain why they were used. Please note that such variables should not be used as proxies for other socially constructed/relevant variables (for example, race or ethnicity should not be used as a proxy for socioeconomic status).*<br>*Provide clear definitions of the relevant terms used, how they were provided (by the participants/respondents, the researchers, or third parties), and the method(s) used to classify people into the different categories (e.g. self-report, census or administrative data, social media data, etc.)*<br>*Please provide details about how you controlled for confounding variables in your analyses.*
Population characteristics	*Describe the covariate-relevant population characteristics of the human research participants (e.g. age, genotypic information, past and current diagnosis and treatment categories). If you filled out the behavioural & social sciences study design questions and have nothing to add here, write "See above."*
Recruitment	*Describe how participants were recruited. Outline any potential self-selection bias or other biases that may be present and how these are likely to impact results.*
Ethics oversight	*Identify the organization(s) that approved the study protocol.*

Note that full information on the approval of the study protocol must also be provided in the manuscript.

Field-specific reporting

Please select the one below that is the best fit for your research. If you are not sure, read the appropriate sections before making your selection.

☒ Life sciences ☐ Behavioural & social sciences ☐ Ecological, evolutionary & environmental sciences

For a reference copy of the document with all sections, see nature.com/documents/nr-reporting-summary-flat.pdf

Life sciences study design

All studies must disclose on these points even when the disclosure is negative.

Sample size	The minimal sample size was determined by a power analysis using PS Power and Sample Size Calculations programm V3.1.6 from Vanderbilt university (http://biostat.mc.vanderbilt.edu/PowerSampleSize) with the following assumptions: We are planning a study of a continuous response variable from independent control and experimental subjects with 1 control per experimental subject. In a previous study the response within each subject group was normally distributed with standard deviation 0.1. If the true difference in the experimental and control means is 0.2, we will need to study 5 experimental subjects and 5 control subjects to be able to reject the null hypothesis that the population means of the experimental and control groups are equal with probability (power) 0.8. The Type I error probability associated with this test of this null hypothesis is 0.05.
Data exclusions	We did not exclude data to reflect the biological variability in our transgenic mice, which is also seen in patients suffering from BAG3P209L myofibrillar myopathy.
Replication	The reproducibility of the experimental findings was verified by using at least n=3 mice for all experiments. All attemps at replication were successful. For experiments not involving mice at least 3 biological replicates and 2 technical replicates were used with similar results.
Randomization	Transgenic mice and controls were randomly assigned to their respective groups. Mice were age matched but not sex matched or littermate matched. For experiments not involving mice biological replicates were randomly assigned to their respective groups.
Blinding	The investigators were blinded to the gene therapy approach during the application of AAV. The researchers were not blinded to group allocation because the difference in size between transgenic mice and controls was too obvious.

Reporting for specific materials, systems and methods

We require information from authors about some types of materials, experimental systems and methods used in many studies. Here, indicate whether each material, system or method listed is relevant to your study. If you are not sure if a list item applies to your research, read the appropriate section before selecting a response.

Materials & experimental systems

n/a	Involved in the study	
☐	☒	Antibodies
☐	☒	Eukaryotic cell lines
☒	☐	Palaeontology and archaeology
☐	☒	Animals and other organisms
☒	☐	Clinical data
☒	☐	Dual use research of concern
☒	☐	Plants

Methods

n/a	Involved in the study	
☒	☐	ChIP-seq
☒	☐	Flow cytometry
☒	☐	MRI-based neuroimaging

Antibodies

Antibodies used

Anti-α-Actinin / ACTA2 (Sigma Aldrich A7811, lot# 027M4813V), used at 1:400 IF
Anti-alpha B Crystallin / CRYAB (Enzo Life Science ADI-SPA-223D), used at 1:5000 WB
Anti-Bag3 (Proteintech 10599-1-AP, lot# 00097899), used at 1:5000 WB; 1:250 IF
Anti-CD45 (Merck Millipore 05-1416, lot# 2854213), used at 1:400 IF
Anti-Desmin (Dako M0760, lot# 7254), used at 1:100 IF
Anti-Filamin C (Biogenes), used at 1:1000 IF
Anti-HSPB6 (Abcam Ab184161, lot#GR156734-8), used at 3:5000 WB
Anti-HSPB7 (Abcam Ab150390, lot# GR105863-7), used at 1:1000 WB
Anti-HSPB8 (Abcam Ab151552, lot# 1001711-5), used at 1:1000 WB
Anti-LC3B (Thermo Fisher Scientific 70012, lot# 900429A), used at 1:1000 WB
Anti-mCherry (Novus Biologicals NBP2-25157, lot# 205091119), used at 1:1000 WB
Anti-p62 / SQSTM1 (Progen GP62-C, lot# FAK24247-01), used at 1:1000 WB, 1:250 IF
Anti-Myc-tag (Abcam, Cat. No.: ab9106 , lot# GR130480-29), used at 1:500 IF
Anti-HA-tag (Proteintech, Cat. No.: 51064-2-AP , lot# 00149369), used at 1:50 IF
Anti-FLAG-tag (Proteintech, Cat. No.: 20543-1-AP , lot# 00169569), used at 1:200 IF
Anti-Pink1 (Proteintech, Cat. No.: 23274-1-AP, lot# 00140989), used at 1:1000 WB; 1:100 IF
BF-F3 / MHCII (Developmental Studies hybridoma Bank, Cat. No.: AB_2266724), used at 1:400 IF
BA-F8 / MHCI (Developmental Studies hybridoma Bank, Cat. No.: AB_10572253), used at 1:400 IF
SC-71 / MHCIIa (Developmental Studies hybridoma Bank, Cat. No.: AB_2147165), used at 1:400 IF
PAX7 (Developmental Studies hybridoma Bank, Cat. No.: AB_528428), used at 1:250 IF
Anti-eGFP (Living Colors (JL-8), Cat. No.: 632381, lot# A5033481), used at 1:1000 WB
Anti-Wheat Germ Agglutinin (WGA), Rodamine (Vector Laboratories, Cat. No.: RL1022, lot# X0712), used at 1:400 IF
Anti-CD68 (eBioscience 14-0681-82, clone FA-11, lot# 4330963), used at 1:100 IF
Anti-cCasp3 (Cell Signaling Technology Cat. No.: 9661S, lot# 47), used at 1:50 IF
Anti-Synpo2 (Sigma Aldrich M9818 Cat. No.: SAB3500586, lot# 60511101), used at 1:500 WB

Anti-GAPDH (Calbiochem CB1001, clone 6C5, lot# 3167011), used at 1:1000 WB
Anti-beta-actin (Invitrogen MA1-140, lot #SE253380), used at 1:2000 WB
Alexa Fluor 647 donkey anti-rat [IgG] (Jackson Immuno Research, Cat. No.: 712-605-153, lot# 123725), used at 1:400 IF
Alexa Fluor 647 donkey anti-mouse [IgG] (Jackson Immuno Research, Cat. No.: 715-605-151, lot# 164103), used at 1:400 IF
Alexa Fluor 647 goat anti-mouse [IgG1] (Jackson Immuno Research, Cat. No.: 115-605-205, lot# 164753), used at 1:400 IF
DyLightTM405 donkey anti-mouse [IgM] (Jackson Immuno Research, Cat. No.: 715-475-140, lot# 115832), used at 1:400 IF
Alexa Fluor 647 goat anti-mouse [IgG2a] (Jackson Immuno Research, Cat. No.: 115-605-206, lot# 148123), used at 1:400 IF
Alexa Fluor 647 donkey anti-rabbit [IgG] (Jackson Immuno Research, Cat. No.: 711-605-152, lot# 167518), used at 1:400 IF, 1:3000 WB
Alexa Fluor 647 donkey anti-guinea pig [IgG] (Jackson Immuno Research, Cat. No.: 706-605-148, lot# 135631), used at 1:400 IF, 1:3000 WB
Cy3 donkey anti-rabbit [IgG] (Jackson Immuno Research, Cat. No.: 711-165-152, lot# 152679), used at 1:400 IF, 1:3000 WB
Cy3 donkey anti-mouse [IgG] (Jackson Immuno Research, Cat. No.: 715-165-151, lot# 162530l), used at 1:400 IF
Alexa Fluor 488 donkey anti-rabbit [IgG] (Jackson Immuno Research, Cat. No.: 711-545-152, lot# 159921), used at 1:400 IF, 1:3000 WB
Cy2 donkey anti-chicken [IgG] (Jackson Immuno Research, Cat. No.: 703-225-155, lot# 168728), used at 1:400 IF
Horseradish Peroxidase-coupled goat anti-rabbit [IgG] (SIGMA, Cat. No. A0545, lot# 124K4820), used at 1:10000 WB
Horseradish Peroxidase-coupled goat anti-guinea pig [IgG] (Millipore, Cat. No. AQ108P, lot# 3032748), used at 1:10000 WB
Horseradish Peroxidase-coupled goat anti-mouse [IgG] (SIGMA, Cat. No. A2304, lot# 065M4761V), used at 1:10000 WB

Validation

Anti-α-Actinin / ACTA2 (Sigma Aldrich A7811, lot# 027M4813V), has been validated for use in immunohistochemistry in mouse tissue, as indicated on the manufacturer's product page.
Anti-alpha B Crystallin / CRYAB (Enzo Life Science ADI-SPA-223D), has been validated for use in western blotting in mouse tissue, as indicated on the manufacturer's product page.
Anti-Bag3 (Proteintech 10599-1-AP, lot# 00097899), has been validated for use in immunohistochemistry and western blotting in mouse tissue, as indicated on the manufacturer's product page.
Anti-CD45 (Merck Millipore 05-1416, lot# 2854213), has been validated for use in immunohistochemistry in mouse tissue, as indicated on the manufacturer's product page.
Anti-Desmin (Dako M0760, lot# 7254), has been validated for use in immunohistochemistry in mouse tissue, as indicated on the manufacturer's product page.
Anti-Filamin C (Biogenes), has been validated in the following publication: PMID: 20060297.
Anti-HSPB6 (Abcam Ab184161, lot#GR156734-8), has been validated for use in western blotting in rat and human tissue , as indicated on the manufacturer's product page. Validation in mice tissue in PMID: 28737513.
Anti-HSPB7 (Abcam Ab150390, lot# GR105863-7), has been validated for use in western blotting in mouse tissue, as indicated on the manufacturer's product page.
Anti-HSPB8 (Abcam Ab151552, lot# 1001711-5), has been validated for use in western blotting in mouse tissue, as indicated on the manufacturer's product page.
Anti-LC3B (Thermo Fisher Scientific 70012, lot# 900429A), has been validated for use in western blotting in mouse tissue, as indicated on the manufacturer's product page.
Anti-mCherry (Novus Biologicals NBP2-25157, lot# 205091119), has been validated for use in western blotting in mouse tissue, as indicated on the manufacturer's product page.
Anti-p62 / SQSTM1 (Progen GP62-C, lot# FAK24247-01), has been validated for use in immunohistochemistry and western blotting in mouse tissue, as indicated on the manufacturer's product page.
Anti-Myc-tag (Abcam, Cat. No.: ab9106 , lot# GR130480-29), has been validated for use in western blotting to detect and purify Myc-tagged proteins expressed in mammalian and non-mammalian cell lines, as indicated on the manufacturer's product page.
Anti-HA-tag (Proteintech, Cat. No.: 51064-2-AP , lot# 00149369), has been validated for use in western blotting to detect and purify HA-tagged proteins expressed in mammalian and non-mammalian cell lines, as indicated on the manufacturer's product page.
Anti-FLAG-tag (Proteintech, Cat. No.: 20543-1-AP , lot# 00169569), has been validated for use in western blotting to detect and purify FLAG-tagged proteins expressed in mammalian and non-mammalian cell lines, as indicated on the manufacturer's product page.
Anti-Pink1 (Proteintech, Cat. No.: 23274-1-AP, lot# 00140989), has been validated for use in immunohistochemistry and western blotting in mouse tissue, as indicated on the manufacturer's product page.
BF-F3 / MHCII (Developmental Studies hybridoma Bank, Cat. No.: AB_2266724), has been validated for use in immunohistochemistry in mouse tissue, as indicated on the manufacturer's product page.
BA-F8 / MHCI (Developmental Studies hybridoma Bank, Cat. No.: AB_10572253), has been validated for use in immunohistochemistry in mouse tissue, as indicated on the manufacturer's product page.
SC-71 / MHCIIa (Developmental Studies hybridoma Bank, Cat. No.: AB_2147165), has been validated for use in immunohistochemistry in mouse tissue, as indicated on the manufacturer's product page.
PAX7 (Developmental Studies hybridoma Bank, Cat. No.: AB_528428), has been validated for use in immunohistochemistry in mouse tissue, as indicated on the manufacturer's product page.
Anti-eGFP (Living Colors (JL-8), Cat. No.: 632381, lot# A5033481), has been validated for use in western blotting in mouse tissue, as indicated on the manufacturer's product page.
Anti-Wheat Germ Agglutinin (WGA), Rodamine (Vector Laboratories, Cat. No.: RL1022), has been validated for use in immunohistochemistry in mouse tissue, as indicated on the manufacturer's product page.
Anti-CD68 (eBioscience 14-0681-82, clone FA-11, lot# 4330963), has been validated for use in immunohistochemistry in mouse tissue, as indicated on the manufacturer's product page.
Anti-cCasp3 (Cell Signaling Technology Cat. No.: 9661S, lot# 47), has been validated for use in immunohistochemistry in mouse tissue, as indicated on the manufacturer's product page.
Anti-Synpo2 (Sigma Aldrich M9818 Cat. No.: SAB3500586, lot# 60511101), has been validated for use in immunohistochemistry in rat tissue, as indicated on the manufacturer's product page.
Anti-GAPDH (Calbiochem CB1001, clone 6C5, lot# 3167011), has been validated for use in western blotting in mouse tissue, as indicated on the manufacturer's product page.
Anti-beta-actin (Invitrogen MA1-140, lot #SE253380), has been validated for use in western blotting in mouse tissue, as indicated on the manufacturer's product page.
Alexa Fluor 647 donkey anti-rat [IgG] (Jackson Immuno Research, Cat. No.: 712-605-153, lot# 123725), has been validated for use in immunohistochemistry in mouse tissue, as indicated on the manufacturer's product page.

Alexa Fluor 647 donkey anti-mouse [IgG] (Jackson Immuno Research, Cat. No.: 715-605-151, lot# 164103), has been validated for use in immunohistochemistry in mouse tissue, as indicated on the manufacturer's product page.
Alexa Fluor 647 goat anti-mouse [IgG1] (Jackson Immuno Research, Cat. No.: 115-605-205, lot# 164753), has been validated for use in immunohistochemistry in mouse tissue, as indicated on the manufacturer's product page.
DyLightTM405 donkey anti-mouse [IgM] (Jackson Immuno Research, Cat. No.: 715-475-140, lot# 115832), has been validated for use in immunohistochemistry in mouse tissue, as indicated on the manufacturer's product page.
Alexa Fluor 647 goat anti-mouse [IgG2a] (Jackson Immuno Research, Cat. No.: 115-605-206, lot# 148123), has been validated for use in immunohistochemistry in mouse tissue, as indicated on the manufacturer's product page.
Alexa Fluor 647 donkey anti-rabbit [IgG] (Jackson Immuno Research, Cat. No.: 711-605-152, lot# 167518), has been validated for use in immunohistochemistry and western blotting in mouse tissue, as indicated on the manufacturer's product page.
Alexa Fluor 647 donkey anti-guinea pig [IgG] (Jackson Immuno Research, Cat. No.: 706-605-148, lot# 135631), has been validated for use in immunohistochemistry and western blotting in mouse tissue, as indicated on the manufacturer's product page.
Cy3 donkey anti-rabbit [IgG] (Jackson Immuno Research, Cat. No.: 711-165-152, lot# 152679), has been validated for use in immunohistochemistry and western blotting in mouse tissue, as indicated on the manufacturer's product page.
Cy3 donkey anti-mouse [IgG] (Jackson Immuno Research, Cat. No.: 715-165-151, lot# 162530l), has been validated for use in immunohistochemistry in mouse tissue, as indicated on the manufacturer's product page.
Alexa Fluor 488 donkey anti-rabbit [IgG] (Jackson Immuno Research, Cat. No.: 711-545-152, lot# 159921), has been validated for use in immunohistochemistry and western blotting in mouse tissue, as indicated on the manufacturer's product page.
Cy2 donkey anti-chicken [IgG] (Jackson Immuno Research, Cat. No.: 703-225-155, lot# 168728), has been validated for use in immunohistochemistry in mouse tissue, as indicated on the manufacturer's product page.
Horseradish Peroxidase-coupled goat anti-rabbit [IgG] (SIGMA, Cat. No. A0545, lot# 124K4820), has been validated for use in western blotting in mouse tissue, as indicated on the manufacturer's product page.
Horseradish Peroxidase-coupled goat anti-guinea pig [IgG] (Millipore, Cat. No. AQ108P, lot# 3032748), has been validated for use in western blotting in mouse tissue, as indicated on the manufacturer's product page.
Horseradish Peroxidase-coupled goat anti-mouse [IgG] (SIGMA, Cat. No. A2304, lot# 065M4761V), has been validated for use in western blotting in mouse tissue, as indicated on the manufacturer's product page.

Eukaryotic cell lines

Policy information about cell lines and Sex and Gender in Research

Cell line source(s)	The study did not include cell lines.
Authentication	*Describe the authentication procedures for each cell line used OR declare that none of the cell lines used were authenticated.*
Mycoplasma contamination	*Confirm that all cell lines tested negative for mycoplasma contamination OR describe the results of the testing for mycoplasma contamination OR declare that the cell lines were not tested for mycoplasma contamination.*
Commonly misidentified lines (See ICLAC register)	*Name any commonly misidentified cell lines used in the study and provide a rationale for their use.*

Animals and other research organisms

Policy information about studies involving animals; ARRIVE guidelines recommended for reporting animal research, and Sex and Gender in Research

Laboratory animals	We used male and female 2-6 weeks old transgenic PGK-Cre/CAG-flox-hBAG3WT-eGFP, and PGK-Cre/CAG-flox-hBAG3P209L-eGFP and littermate control mice (Mus musculus) a mixed 129S6/SvEvTac x C57BL/6Ncr x CD-1 genetic background.
Wild animals	The study did not involve wild animals.
Reporting on sex	This information has not been collected. Due to the small litter size, it was not possible to examine separate groups of each sex.
Field-collected samples	The study did not involve samples collected from the field.
Ethics oversight	Animal experiments were approved by the responsible governmental animal care and use office, the Landesamt für Natur, Umwelt und Verbraucherschutz, LANUV (81-02.04.2019.A062 and 81-02.04.2021.A068)

Note that full information on the approval of the study protocol must also be provided in the manuscript.

Plants

Seed stocks	The study did not involve plants.
Novel plant genotypes	*Describe the methods by which all novel plant genotypes were produced. This includes those generated by transgenic approaches, gene editing, chemical/radiation-based mutagenesis and hybridization. For transgenic lines, describe the transformation method, the number of independent lines analyzed and the generation upon which experiments were performed. For gene-edited lines, describe the editor used, the endogenous sequence targeted for editing, the targeting guide RNA sequence (if applicable) and how the editor was applied.*
Authentication	*Describe any authentication procedures for each seed stock used or novel genotype generated. Describe any experiments used to assess the effect of a mutation and, where applicable, how potential secondary effects (e.g. second site T-DNA insertions, mosiacism, off-target gene editing) were examined.*
